# PIP_2_ promotes the incorporation of CD43, PSGL-1, and CD44 into nascent HIV-1 particles

**DOI:** 10.1126/sciadv.ads9711

**Published:** 2025-04-04

**Authors:** Ricardo de Souza Cardoso, Tomoyuki Murakami, Binyamin Jacobovitz, Sarah L. Veatch, Akira Ono

**Affiliations:** ^1^ Department of Microbiology and Immunology, University of Michigan Medical School, Ann Arbor, MI, USA.; ^2^ BRCF Microscopy Core, University of Michigan Medical School, Ann Arbor, MI, USA.; ^3^ Department of Biophysics, University of Michigan, Ann Arbor, MI, USA.

## Abstract

Determinants regulating sorting of host transmembrane proteins at sites of enveloped virus assembly on the plasma membrane (PM) remain poorly understood. Here, we demonstrate that the PM acidic phospholipid phosphatidylinositol 4,5-bisphosphate (PIP_2_) regulates this sorting into an enveloped virus, HIV-1. Incorporation of CD43, PSGL-1, and CD44 into HIV-1 particles has profound effects on viral spread; however, the mechanisms promoting their incorporation were unknown. We found that depletion of cellular PIP_2_ blocks incorporation of CD43, PSGL-1, and CD44 into HIV-1 particles. Expansion microscopy revealed that PIP_2_ depletion diminishes nanoscale coclustering between viral structural protein Gag and the three transmembrane proteins at the PM and that Gag induces PIP_2_ enrichment at its vicinity. CD43, PSGL-1, and CD44 also increased local PIP_2_ density, revealing their PIP_2_ affinity. Together, these results support a previously unknown mechanism where local enrichment of an acidic phospholipid drives coclustering between viral structural and cellular transmembrane proteins, thereby modulating the content, and hence the fate, of progeny virus particles.

## Teaser

The acidic phospholipid PIP_2_ attracts host transmembrane proteins that modulate HIV-1 spread into assembling HIV-1 particles.

## INTRODUCTION

Enveloped viruses that assemble at the cell surface often incorporate cellular transmembrane proteins (*1*–*4*), which can either facilitate or prevent the viral spread (*4*–*6*). The incorporation of these proteins into viruses is determined by their distribution relative to the viral assembly sites at the cell surface. Among the factors that influence plasma membrane (PM) distribution of the cellular transmembrane proteins include interactions with other proteins, association with lipids or lipid nanodomains, endocytosis and exocytosis, diffusion barriers formed by PM-associated proteins, and membrane curvature. Notably, these factors can also be modulated by virus assembly.

Among the viruses that assemble at the PM is HIV-1 (*7*–*9*). The HIV-1 assembly is governed by the structural polyprotein Gag. Gag binds the PM through its N-terminal myristoylation and a highly basic region (HBR) in the MA domain (MA-HBR) (*7*, *10*). The MA-HBR was shown in multiple in vitro studies to interact with phosphatidylinositol 4,5-bisphosphate (PIP_2_) (*11*–*13*), a negatively charged acidic phospholipid enriched at the PM (*14*). HIV-1 particle assembly affects the distribution of diverse cellular transmembrane proteins by recruiting or excluding them from the assembly sites (*15*, *16*). The recruitment of some proteins (e.g., tetraspanin CD81 and tetherin, an antiviral protein) into HIV-1 assembly sites relies on membrane curvature (*15*, *17*, *18*), whereas some other proteins are incorporated into HIV-1 due to their association with cholesterol-enriched membrane microdomains, which coincide with virus assembly sites (*4*, *18*–*21*).

In polarized CD4^+^ T cells, HIV-1 Gag proteins accumulate to the rear-end protrusion called uropod (*22*). We have previously shown by total internal reflection fluorescence (TIRF)–based nanoscopy approaches that three uropod-localizing transmembrane proteins, CD43, PSGL-1, and CD44, cocluster with Gag at the PM of HeLa and T cells (*23*). These three transmembrane proteins participate in the cell-cell adhesion processes (*24*). The presence of CD43 and PSGL-1 in HIV-1 particles impairs the attachment of the virion to the target cells (*25*–*27*), while the presence of CD44 on HIV-1 particles promotes trans-infection of CD4^+^ T cells mediated by lymph node stromal cells (*5*, *28*). While it has been reported that HIV-1 proteins Vpu and Nef play a role in the reduction of cellular PSGL-1 levels (*26*, *27*), virus-induced down-regulation has not been mechanistically described for CD43 (*29*). Despite their effects on HIV-1 spread, the mechanisms underlying the incorporation of these three host proteins into HIV-1 remain unknown.

Our past study found that coclustering between Gag and CD43, PSGL-1, and CD44 requires both the juxtamembrane polybasic sequences (JMPBSs) of these three transmembrane proteins and the MA-HBR of Gag proteins (*23*). As both JMPBS and MA-HBR protein regions are positively charged, we hypothesize that their interaction is mediated by PIP_2_, a highly negatively charged lipid resident to the PM inner leaflet. Consistent with this hypothesis, previous studies have shown that HIV-1 Gag can reduce the mobility of PIP_2_ at HIV-1 assembly sites and that PIP_2_ is enriched in the released virus particles (*30*–*32*). However, it remains unknown whether a Gag-engaged PIP_2_ at HIV-1 assembly sites plays roles beyond anchoring Gag to the PM and, if so, how these roles affect the virus assembly process. In the current study, we demonstrate that PIP_2_ is enriched near Gag and that PIP_2_ facilitates the enrichment of CD43, PSGL-1, and CD44 near Gag at the PM and the incorporation of these proteins into released particles. Our results therefore reveal that PIP_2_ plays roles in HIV-1 assembly beyond the Gag-PM binding, namely, in the recruitment of host proteins that regulate virus spread into nascent virus particles.

## RESULTS

### PIP_2_ depletion reduces the incorporation of CD43, PSGL-1, and CD44 into HIV-1 particles

Previous studies demonstrated that the nanoscale colocalization of CD43, PSGL-1, and CD44 with Gag at the ventral PM (*23*) is dependent on both the MA-HBR of Gag and the JMPBS of these three transmembrane proteins. To determine the roles for JMPBS and PIP_2_ in HIV-1 incorporation of the three transmembrane proteins, we generated HIV-1 virus-like particles (VLPs) from HeLa cells with perturbed PIP_2_ levels and probed the incorporation of these proteins into isolated VLPs. HeLa cells were chosen because they naturally lack endogenous expression of CD43 and PSGL-1 and because CD44-depleted HeLa cells were available (*28*). In addition, the expression of HIV-1 in HeLa cells via plasmid transfection is a well-established model system for studying HIV-1 assembly using both biochemical and microscopy approaches. We transfected HeLa cells with three plasmids. The first plasmid was an HIV-1 molecular clone encoding the Gag protein with its N terminus fused to the 10-residue N-terminal sequence of Fyn kinase [Fyn(10)/Gag]. In this construct, the single N-myristoylation signal of wild-type (WT) Gag is replaced with a triple acylation, enabling Gag membrane binding and subsequent virus release even in the absence of PIP_2_ (*13*). Our previous studies showed that upon PIP_2_ depletion, WT Gag fails to bind the PM, with the majority remaining in the cytosol, leading to ~10-fold reduction of virus particle production. In contrast, in the absence of PIP_2_, a large part of Fyn(10)/Gag undergoes PM binding with a subpopulation mislocalized to intracellular compartments, resulting in a substantial but not complete rescue of particle production (*13*). The second plasmid encodes WT CD43, PSGL-1, or CD44 or their variants containing three or six alanine substitutions of basic amino acid residues in JMPBS (3A or 6A). The third plasmid is a Tat-inducible plasmid that expresses full-length 5-phosphatase IV (5ptaseIV FL) or its inactive variant 5ptaseIV Δ1 upon expression of HIV-1 genes. Expression of 5ptaseIV FL depletes cellular PIP_2_ and PIP_3_ (*33*), although the steady-state level of PIP_3_ is negligible relative to PIP_2_ (*34*).

**Fig. 1.**
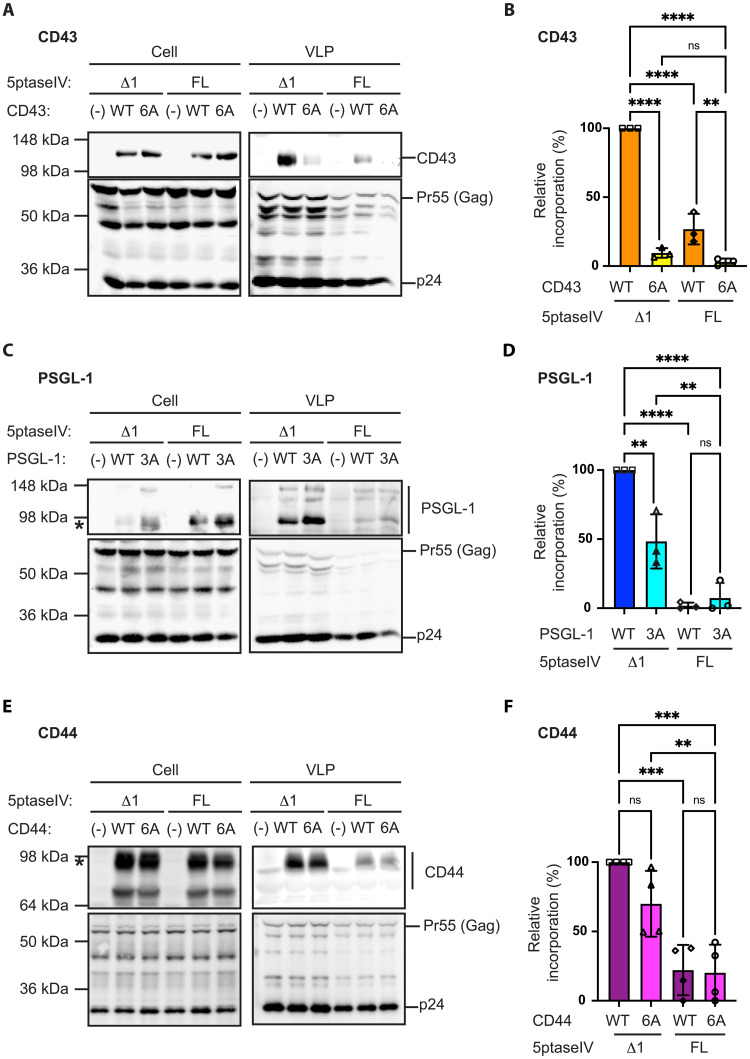
PIP_2_ depletion diminishes incorporation of CD43, PSGL-1, and CD44 into VLPs. HeLa cells were transfected with plasmids encoding Fyn(10)/Gag, indicated host transmembrane proteins or their variants lacking native JMPBS, and 5ptaseIV Δ1 or FL. Western blotting analysis of cell and viral lysates was performed for CD43 (**A** and **B**), PSGL-1 (**C** and **D**), and CD44 (**E** and **F**) and HIV-1 Gag proteins. Representative blots are shown [(A), (C), and (E)]. The asterisks in (C) and (E) denote the bands for PSGL-1 and CD44 quantitated for (D) and (F), respectively. In (B), (D), and (F), the incorporation efficiency was calculated as the ratio of the indicated host transmembrane proteins in viral lysates versus cell lysates, which was normalized for the amount of released particles represented by p24 in virus lysates. The relative incorporation efficiency for each condition was calculated in comparison to the incorporation efficiency of WT transmembrane proteins into virus in the presence of 5ptaseIV Δ1. The data from three independent experiments are shown. The *P* value was determined using analysis of variance (ANOVA) and one-way Tukey’s multiple comparison test. **P < 0.01; ***P < 0.001; ****P < 0.0001; ns, not significant.

We first investigated the effect of the 3A or 6A mutations in JMPBS on the incorporation of CD43, PSGL-1, and CD44 within Fyn(10)/Gag VLPs in cells transfected with the control plasmid encoding 5ptaseIV Δ1. As observed previously for HIV-1 particles consisting of WT Gag (*23*), amino acid substitutions of JMPBS prevented the incorporation of CD43 into VLPs consisting of Fyn(10)/Gag (Fig. 1, A and B), indicating that the triple acylation in place of N-myristoylation of Gag does not alter the JMPBS dependence of CD43 incorporation into nascent, i.e., newly assembled and released, VLPs. We further observed that changes in the JMPBS reduced the incorporation of PSGL-1 partially (Fig. 1, C and D), whereas similar changes caused no significant reduction in the incorporation of CD44 (Fig. 1, E and F). Consistent with our previous findings, which showed that cellular PSGL-1 levels decrease because of their incorporation into HIV-1 particles that are released extracellularly (*25*), we observed a reduction of WT PSGL-1 but not 3A levels in cells expressing 5ptaseIV ∆1 in most of our independent experimental replicates (Fig. 1C). Together, these results demonstrate that the basic amino acid residues in JMPBS of CD43 and PSGL-1 are important for their incorporation into HIV-1 VLPs, regardless of acylation types present at the Gag N terminus.

Next, we explored how PM PIP_2_ may contribute to the incorporation of the three transmembrane proteins into HIV-1 VLPs. This was accomplished by monitoring the levels of these proteins within nascent VLPs produced by cells expressing 5ptaseIV Δ1 versus 5ptaseIV FL. Expression of 5ptaseIV FL significantly diminished the levels of the three transmembrane proteins CD43, PSGL-1, and CD44 in released particles (Fig. 1). The fold change in the incorporation into HIV-1 VLP upon PIP_2_ depletion was much greater than the change caused by substitutions in JMPBS of PSGL-1 (~20-fold with PIP_2_ depletion versus 2-fold with JMPBS substitutions) and CD44 (~5-fold with PIP_2_ depletion versus no significant reduction with JMPBS substitutions). In our previous study (*23*), we showed that intercellular adhesion molecule–1 (ICAM-1), another transmembrane protein that has been shown to be incorporated into HIV-1 particles (*35*–*37*), does not cocluster with Gag at the ventral PM and that its incorporation into HIV-1 significantly increases when its cytoplasmic tail (CT) is replaced with PSGL-1 CT, suggesting that ICAM-1 incorporation into virus particles occurs in a different mechanism. We found that ICAM-1 incorporation into VLPs was unaffected by 5ptaseIV FL expression (fig. S1, A and B). These results indicate that PIP_2_ depletion specifically impairs the incorporation of CD43, PSGL-1, and CD44 into VLPs but not that of ICAM-1. Notably, flow cytometry analysis of the transmembrane proteins indicated that the presence of 5ptaseIV FL did not significantly reduce both cell surface and overall expression of CD43, PSGL-1, and CD44 in Gag-expressing cells (fig. S2, A to F). The cell viability does not account for the lower levels of CD43, PSGL-1, and CD44 associated with VLPs because we did not observe any significant cytotoxicity effects of PIP_2_ depletion upon expression of 5ptaseIV FL (fig. S2G). We also note that although PIP_2_ regulates the organization of cortical actin cytoskeleton (*14*) involved in various PM processes, treatment with latrunculin B (Lat B), a compound that prevents F-actin formation, did not show any significant effect on viral incorporation of the three transmembrane proteins (fig. S3, A to F). Together, these results indicate that PIP_2_ is a major determinant for the efficient incorporation of CD43, PSGL-1, and CD44 into HIV-1 particles.

### Expansion microscopy allows for detection of HIV-1 assembly sites at higher resolutions

Because PIP_2_ depletion suppresses the incorporation of CD43, PSGL-1, and CD44 into HIV-1 particles without interfering the trafficking of these proteins to the PM, we hypothesized that PIP_2_ depletion instead alters the distribution of these three cellular proteins relative to HIV-1 assembly sites at the PM. To examine the protein distribution in and around particle assembly sites, which are at the order of tens to hundreds of nanometers, we sought to use a super-resolution microscopy method.

**Fig. 2.**
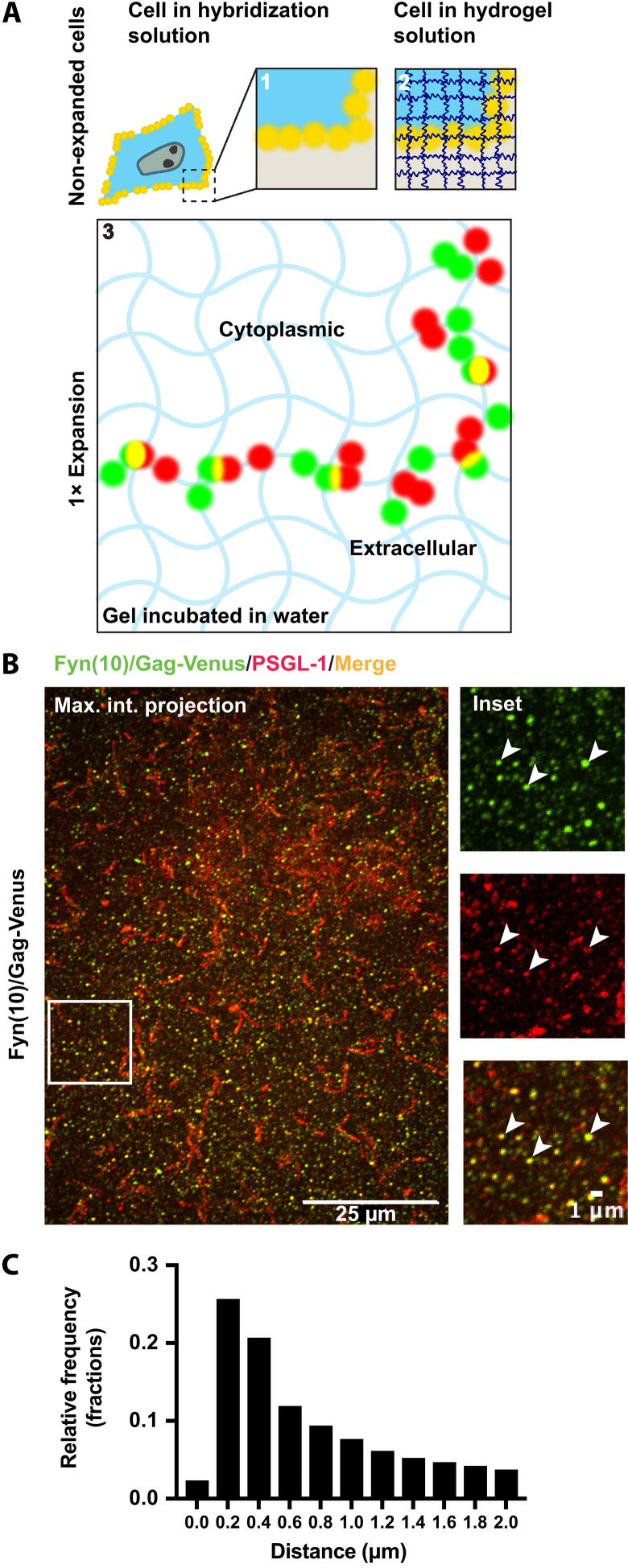
ExM analysis of transmembrane proteins and Gag at the PM. (**A**) Schematic representation of ExM. In step 1, cells are incubated in the hybridization solution, which contains acrylamide and formaldehyde. In step 2, the cells are embedded in acrylamide plus sodium acrylate copolymer hydrogel to which biomolecules are cross-linked. Expansion of hydrogel after incubation in water (step 3) enables the separation of the biomolecules (green and red) above optical resolution limits, which were originally not separable before expansion (yellow dots at steps 1 and 2). (**B**) ExM of HeLa cells transfected with Fyn(10)/Gag-Venus along with PSGL-1. Cells were fixed at 16 to 18 hours post-transfection, immunostained for cell surface–expressed PSGL-1 (red), permeabilized, and immunostained for Gag [with anti–green fluorescent protein (GFP); green] before the ExM procedure. Arrowheads, sites of coclustering between PSGL-1 and Fyn(10)/Gag-Venus. (**C**) Histogram of the distances from Fyn(10)/Gag-Venus spots to their nearest PSGL-1 spots. The expanded immunofluorescence images were acquired with a Nikon Spinning Disk confocal microscope. Magnification, ×100. For image processing and quantification, the Imaris (version 10.0.1) software was used. Scale bars, 25 μm (whole-cell images) and 1 μm (insets).

Stochastic optical reconstruction microscopy (STORM) coupled with TIRF illumination has been used frequently to achieve super-resolution analysis of HIV-1 assembly and its relationship with host proteins such as tetherin (*17*, *38*–*42*). However, because of the sizes of CD43 and PSGL-1 extracellular domains, which reach 45 to 50 nm (*43*, *44*), it was conceivable that TIRF-based approaches, which examine only up to ~100 nm from the coverslip, introduce detection bias (fig. S4A). To overcome this potential limitation and to have a broader comprehension of HIV assembly at not only ventral but also dorsal PMs, we used a recently developed super-resolution technique, expansion microscopy (ExM) (*45*). In ExM, cells are embedded in a hydrogel that swells in an isotropic way in *x*, *y*, and *z* axis in the presence of water. After one round of expansion, the cells increase their sizes between 3.5 and 5.5 times depending on the cell types tested in different studies, which allows analysis using conventional confocal microscopes to achieve a resolution equivalent to ~50 to 60 nm (Fig. 2A) (*46*, *47*). Consistent with the literature, nuclei of cells expanded using the protocol based on M'Saad and Bewersdorf (*47*) were average 4.6 times larger in perimeter length than those of the nonexpanded cells after one round of expansion (fig. S4, B and C). The 4.6-fold expansion was consistently observed in cell populations expanded in independent experiments, suggesting that sample-to-sample variations are small (fig. S4C). Although two rounds of expansion (fig. S4B) yielded a better resolution, it became technically difficult to image the dorsal membrane, which fell outside the working distance of the objective. In addition, one round of the expansion allowed us to distinguish individual clusters of yellow fluorescent protein (YFP)–tagged Fyn(10)/Gag [Fyn(10)/Gag-Venus] and PSGL-1 on the cell surface readily, which cannot be distinguished in nonexpanded cells (fig. S4, D and E). Therefore, in the subsequent experiments, we used the ExM approach with one round of expansion to determine the distribution of proteins and a lipid at the PM of HIV-1–expressing cells.

### Most VenusYFP-tagged Fyn(10)/Gag is within 1 μm from the PM in ExM

To analyze the nanoscale colocalization between Gag and host PM components on the cell surface using the ExM approach, we first identified the population of Gag bound to the PM detected as fluorescent puncta. We note that these fluorescent Gag spots can represent a range of different assembly stages. Although previous studies showed that ~75% of Fyn(10)/Gag-Venus in HeLa cells is found in membrane fractions (compared to 55% for WT Gag) (*48*), the non–membrane-bound Gag population, which is irrelevant to this study, still exists in these cells. To determine the distance from the PM that distinguishes the PM-bound and non–PM-bound Gag populations in ExM, we measured the shortest distances from each Fyn(10)/Gag-Venus signal to the PM. HeLa cells were cotransfected with an HIV-1 molecular clone encoding Fyn(10)/Gag-Venus and a plasmid encoding PSGL-1, which served as the PM marker. After expansion, cells were imaged using confocal microscopy (Fig. 2B), and the images were postprocessed to determine the weighted centroid of the Gag and PSGL-1 signals (see Materials and Methods for details). Then, we measured the distances from each Gag centroid to the nearest PSGL-1 centroid and plotted these distances in the histograms shown in Fig. 2C. We found that the distances from Fyn(10)/Gag-Venus to the nearest PSGL-1 in expanded cells most frequently ranged around 0.2 to 0.4 μm, with ~70% of Gag localized within 1 μm of the nearest PSGL-1 signal, suggesting that Gag signals present within 1 μm from a PM marker in ExM correspond to the membrane-bound population detected in the membrane flotation analyses reported previously (*48*). On the basis of these observations, in the subsequent experiments, we defined the population of Gag spots that are within 0 to 1 μm from the nearest cell surface transmembrane proteins as the PM-bound Gag.

### ExM confirms CT-dependent coclustering of CD43 and PSGL-1 with Gag

**Fig. 3.**
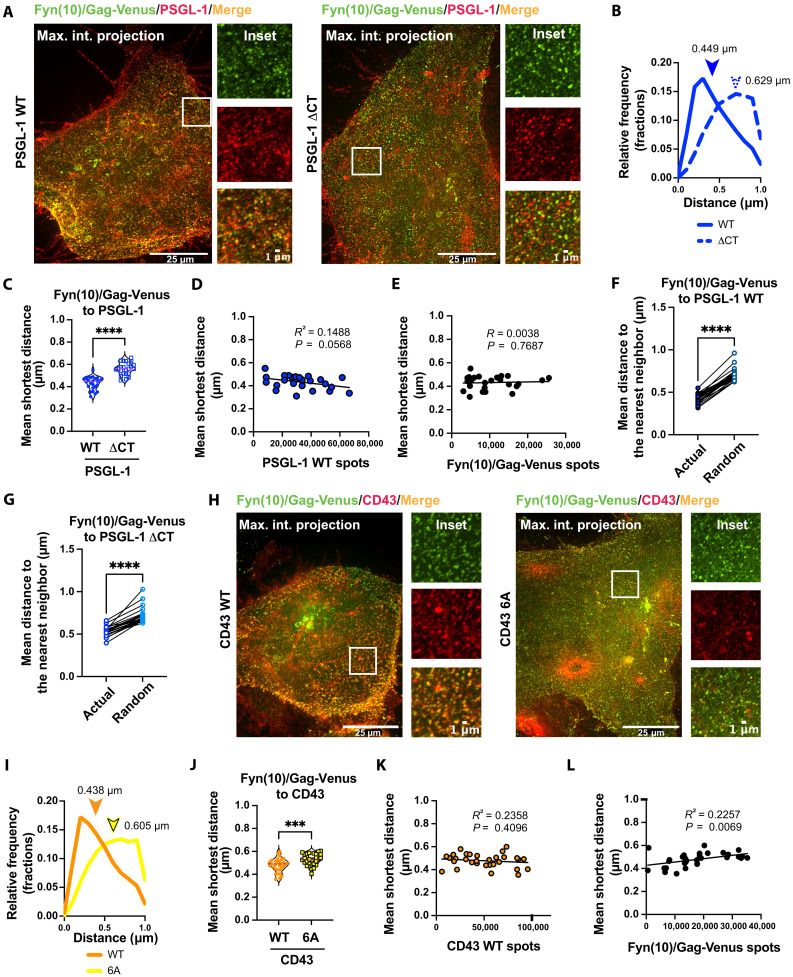
ExM analysis of transmembrane proteins and Gag at the PM. (**A**) ExM of HeLa cells transfected with Fyn(10)/Gag-Venus along with PSGL-1 WT or ΔCT. Cells were fixed, immunostained, and analyzed using the ExM procedure as in Fig. 2. (**B**) Histograms of the distances from Fyn(10)/Gag-Venus to PSGL-1 in the cells shown in (A). Mean shortest distance values for these two cells are shown with arrowheads. (**C**) Mean shortest distances from Fyn(10)/Gag-Venus to PSGL-1 WT and ΔCT. The mean shortest distance from Fyn(10)/Gag-Venus to the indicated host transmembrane protein was calculated for each cell and compiled in single graphs for all cells examined in three independent experiments. (**D** and **E**) Correlations between the shortest distance from Fyn(10)/Gag-Venus to PSGL-1 WT and the number of PSGL-1 or Fyn(10)/Gag-Venus spots are examined. (**F** and **G**) Mean nearest neighbor distances from Fyn(10)/Gag-Venus to PSGL-1 WT or ΔCT are compared between cells with actual and simulated randomized distributions of the two proteins. (**H** to **L**) ExM of cells transfected with Fyn(10)/Gag-Venus and CD43 WT or 6A. The analyses in (I) to (L) were performed as in (B) to (E). All the experiments were repeated at least three times, and 7 to 12 cells for each biological replicate (total of 26 to 33 cells) were analyzed. The *P* value was determined using nonpaired Student’s *t* test [(C) and (J)] or paired *t* test [(F) and (G)]. ***P < 0.001; ****P < 0.0001. *R*^2^ was determined by simple linear regression analysis. For randomizing the locations of fluorescence signals [(F) and (G)], the *x*, *y*, and *z* coordinates for each membrane protein signal were used to determine the cell surface, and then points redistributed randomly on the estimated surface were used to calculate nearest neighbor distances.

Consistent with the incorporation of PSGL-1 in Fyn(10)/Gag VLPs (Fig. 1), colocalization between Fyn(10)/Gag-Venus and PSGL-1 was readily visible (Fig. 2B, white arrowheads). However, coclustering between Gag and cellular transmembrane proteins at nanoscales may not necessarily be detected as exactly overlapping signals in ExM. To quantitate the degree of coclustering, we measured the shortest distances between the PM-bound Gag and cellular transmembrane proteins. To validate this approach, we repeated several past measurements conducted in TIRF-STORM using ExM. We previously demonstrated that deletion of the cytoplasmic domain of PSGL-1 (PSGL-1 ΔCT) and basic-to-neutral amino acid substitutions in CD43 JMPBS (CD43 6A) almost completely diminished coclustering of these proteins with Gag at the ventral membrane of HeLa cells (*23*). Because of this distinct phenotype in coclustering with Gag compared with their WT counterparts, we chose these two variants for validating our ExM approach. We transfected HeLa cells with Fyn(10)/Gag-Venus and PSGL-1, CD43, or their variants and analyzed the cells by ExM (Fig. 3, A and H). For quantitation of coclustering between Gag and the transmembrane proteins in ExM, we determined the distances from PM-bound Gag to the nearest transmembrane proteins at the PM (shortest distance) for each cell analyzed by ExM (Fig. 3, B and I). We then calculated the mean of these distances for each cell [arrowheads in Fig. 3 (B and I) denote the means for the examples] and compared the ranges of the mean shortest distances between the experimental conditions (Fig. 3, C and J). Notably, we confirmed that the presence of the Fyn(10) modification at the Gag N terminus does not affect the shortest distances from Gag to PSGL-1 (fig. S5, A to C).

Consistent with past work, we observed that the mean shortest distances from Gag to PSGL-1 ΔCT are longer than those to WT PSGL-1 (Fig. 3C). Likewise, the basic-to-neutral amino acid changes in CD43 CT JMPBS increased the shortest distances from Gag to CD43 6A compared to WT CD43 (Fig. 3J). We note that the differences in the mean shortest distances tend to be smaller than the differences in the peak (most frequent) shortest distances (Fig. 3, B and I). We additionally determined the Pearson’s correlation coefficient using the maximum intensity projection images based on the *z*-stacks of the same microscopy images analyzed by the shortest distance method. In good accordance with shortest distance measurements, Pearson’s coefficient showed that the CT of PSGL-1 promotes its colocalization with Gag (fig. S4F). Nonetheless, we chose the shortest distance approach over Pearson’s correlation coefficient analysis for coclustering between Gag and host molecules at the PM. Pearson’s correlation coefficient analysis uses two-dimensional (2D) maximum intensity projection images, which can obscure correlations in the 3D space by projecting dorsal and ventral membranes onto the same plane. In addition, because Gag exists both at the PM and in the cytoplasm, the maximum projection images do not represent the Gag distribution on the PM alone. In contrast, the shortest distance approach avoids these issues by specifically measuring distances between Gag and host molecules both at the PM, providing a more accurate and reliable analysis of coclustering.

It is conceivable that the decrease in the shortest distance (which is interpreted here as increased coclustering) could correlate with the abundances of the proteins at the PM; however, across the range of surface expression levels observed under the experimental conditions we used, the shortest distance from Fyn(10)/Gag-Venus to PSGL-1 has no strong correlation with the numbers of PSGL-1 or Fyn(10)/Gag-Venus spots (Fig. 3, D and E, respectively). This was also the case with the shortest distances from Fyn(10)/Gag-Venus to CD43 (Fig. 3, K and L). We further sought to test in a different approach whether the shortest distance observed above simply reflects the densities of Gag and PSGL-1 or whether it shows bona fide coclustering. To this end, we compared nearest neighbor distances between Gag and PSGL-1 in actual and randomized distribution over the same cell surfaces. This comparison showed that the nearest neighbor distances from Fyn(10)/Gag-Venus to PSGL-1 WT are shorter in actual than in randomized distribution (Fig. 3F). The same was observed with the distances from Fyn(10)/Gag-Venus to PSGL-1 ΔCT (Fig. 3G). On the basis of these results, we concluded that the shortest distances measured in Fig. 3 (C and J) reflect their colocalization rather than the relative abundances of the two proteins of interest (e.g., Gag and PSGL-1). Together, these results demonstrate that ExM allows for nanoscale analyses of coclustering between two proteins at not only ventral but also entire plasma membranes.

### PIP_2_ depletion increases the distances from Gag to the cellular transmembrane proteins at the PM

**Fig. 4.**
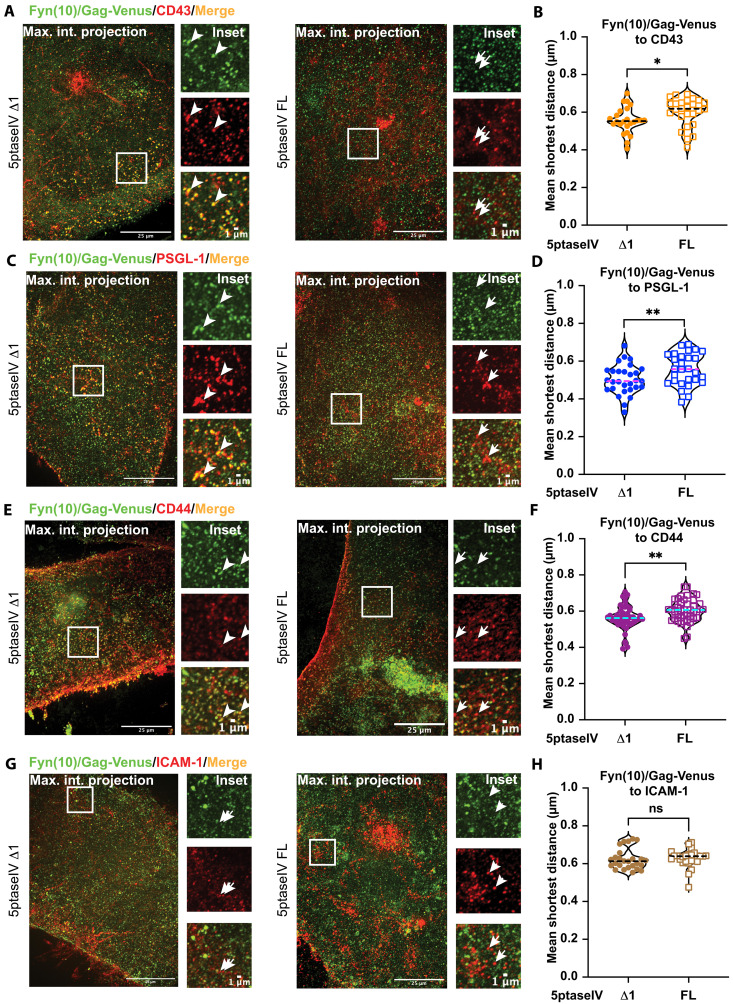
PIP_2_ depletion decreases coclustering of Fyn(10)/Gag-Venus with CD43, PSGL-1, and CD44, but not ICAM-1. (**A**, **C**, **E**, and **G**) ExM of HeLa cells transfected with plasmids encoding Fyn(10)/Gag-Venus (green), the indicated host transmembrane proteins (red), and 5ptaseIV Δ1 or FL. Cells were fixed, immunostained, and expanded as in Fig. 2. The insets correspond to regions shown in white boxes in maximum intensity projection images. Arrowheads, sites of coclustering between Fyn(10)/Gag-Venus and the corresponding cellular transmembrane protein. Arrows, sites where there is no coclustering of the transmembrane proteins with Fyn(10)/Gag-Venus. (**B**, **D**, **F**, and **H**) Mean shortest distances from Fyn(10)/Gag-Venus to the indicated host transmembrane proteins are compared between the presence of 5ptaseIV Δ1 and FL. The experiments were repeated three to six times, and at least 5 to 12 cells from each biological replicate were analyzed (total of 24 to 47 cells). The *P* value was determined using Student’s *t* test. *P < 0.05; **P < 0.01; ns, not significant. Scale bars, 25 μm (whole-cell images) and 1 μm (insets). Image acquisition, processing, and quantification were performed as in Fig. 3.

Using the ExM-based approach validated above, we next tested the hypothesis that PIP_2_ promotes the coclustering between Gag and CD43, PSGL-1, and CD44 at the PM of HIV-1–expressing cells. HeLa cells were transfected with a molecular clone encoding Fyn(10)/Gag-Venus and plasmids encoding the cellular transmembrane proteins along with plasmids encoding 5ptaseIV Δ1 or FL. Coclustering between Fyn(10)/Gag-Venus and CD43, PSGL-1, or CD44 was analyzed as in Fig. 3 (C and J). We found that expression of 5ptaseIV FL caused significant increases in the distances from Fyn(10)/Gag-Venus to CD43 (Fig. 4, A and B), PSGL-1 (Fig. 4, C and D), and CD44 (Fig. 4, E and F). Notably, expression of 5ptaseIV FL did not change the distances from Fyn(10)/Gag-Venus to ICAM-1 (Fig. 4, G and H), which does not specifically cocluster with HIV-1 Gag (*16*). We also tested whether the shortest distances from Fyn(10)/Gag-Venus to the cellular transmembrane proteins correlate with their number of spots (abundance of the proteins of interest) at the PM. In the cases of CD43, PSGL-1, and CD44, we found no correlations in 5ptaseIV Δ1–expressing cells (fig. S6, A to F). In contrast, ICAM-1 showed a moderate correlation between the number of molecules at the cell surface and the association with Gag in cells expressing 5ptaseIV Δ1 (fig. S6, G and H). Together, these results indicate that PIP_2_ promotes coclustering of HIV-1 Gag with CD43, PSGL-1, and CD44 but not with ICAM-1.

### PIP_2_ coclusters with Gag at the PM

**Fig. 5.**
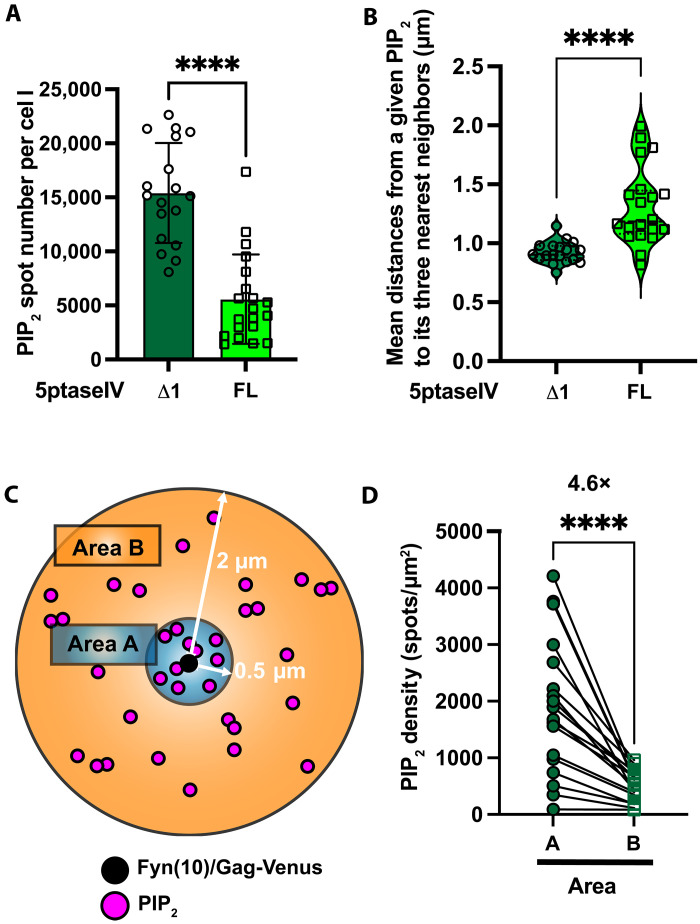
PIP_2_ accumulates at VLP assembly sites at the PM. (**A**) The number of fluorescent spots representing PIP_2_ was determined in HeLa cells cotransfected with plasmids encoding Fyn(10)/Gag-Venus and 5ptaseIV Δ1 or FL. (**B**) Distances from a given PIP_2_ spot to its three nearest neighbors are compared in cells examined in (A). (**C**) Schematic representation of an approach to quantify the accumulation of PIP_2_ to the assembly sites. (**D**) PIP_2_ spots were counted in areas A [within a radius of 0.5 μm from a Fyn(10)/Gag-Venus spot] and B [within a radius of 2 μm from Fyn(10)/Gag-Venus but excluding area A] and normalized for the area size with the approximation that total area B is 15-fold larger than total area A. The experiments were repeated at least three times, and 5 to 9 cells from each biological replicate were analyzed (total of 19 cells). The *P* value was determined using nonpaired Student’s *t* test (A and B) and paired *t* test (D). ****P < 0.0001.

To investigate the mechanism by which PIP_2_ promotes coclustering of Fyn(10)/Gag-Venus with the cellular transmembrane proteins, we sought to determine PIP_2_ localization. We chose to detect PIP_2_ by an immunostaining procedure, which is expected to allow for the detection of the lipid with minimal perturbation (*49*). To validate this approach, 5ptaseIV Δ1– or FL–transfected cells were probed with anti-PIP_2_ and expanded and quantified for PIP_2_ spots under each condition. As expected, the 5ptaseIV FL expression reduced the number of PIP_2_ spots/clusters approximately three times compared to the control in the total cell surface (Fig. 5A). In addition, the expression of 5ptaseIV FL caused an increase in the distances from a given PIP_2_ signal to its three nearest neighbors (Fig. 5B), revealing that PIP_2_ becomes more sparsely distributed when its density decreases with the expression of 5ptaseIV FL. These results indicate that this approach allows for a comparison of PIP_2_ distribution using the ExM approach.

**Fig. 6.**
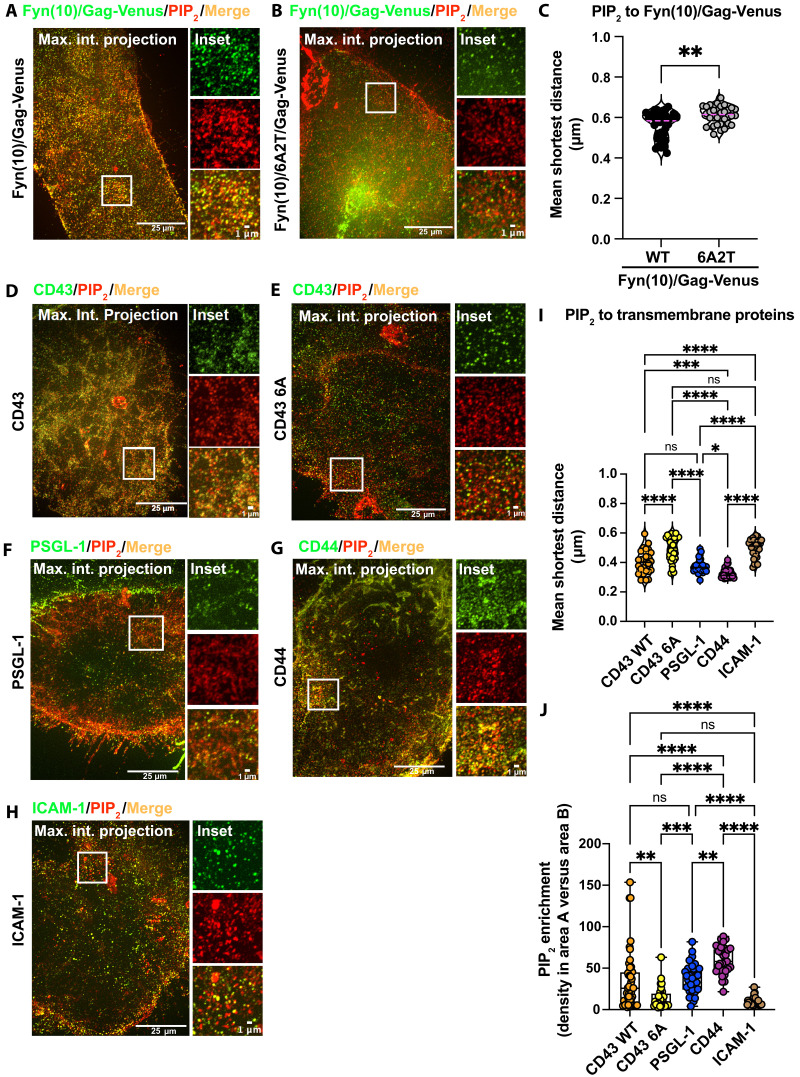
PIP_2_ accumulates to the proximity of Fyn(10)/Gag and cellular transmembrane proteins CD43, PSGL-1, and CD44, but not ICAM-1. (**A** and **B**) HeLa cells expressing either Fyn(10)/Gag-Venus (A) or Fyn(10)/6A2T/Gag-Venus (B) were fixed, probed with anti-PIP_2_ (red) and anti-GFP (for the detection of Gag; green) antibodies, and analyzed by ExM. Note that although the maximum intensity projections for Fyn(10)/Gag-Venus in (A) and (B) display the entire Gag signals, only the PM-associated populations were examined for the quantitative analyses (see fig. S7B for the comparisons between the entire and PM-associated Gag signals). The insets correspond to regions shown in white boxes in the whole-cell images. (**C**) The mean shortest distances from PIP_2_ to Fyn(10)/Gag-Venus and Fyn(10)/6A2T/Gag-Venus were compared. (**D** to **H**) HeLa cells expressing CD43 WT (D), CD43 6A (E), PSGL-1 (F), CD44 (G), or ICAM-1 (H) were probed with anti-PIP_2_ (red) and antibodies for the indicated cellular transmembrane proteins (green). The insets correspond to the boxed areas shown in whole-cell images. (**I**) Quantification of the means of shortest distances from PIP_2_ to each cellular protein. (**J**) PIP_2_ enrichment determined as the PIP_2_ density in area A divided by that in area B. The PIP_2_ densities were calculated with the approximation that total area B is 15-fold larger than total area A. The experiments were repeated three to four times, and at least 9 to 13 cells from each biological replicate were analyzed (total of 28 to 39 cells). The *P* value was determined using Student’s *t* test (C), ANOVA, and one-way Tukey’s multiple comparison test [(I) and (J)]. *P < 0.05; **P < 0.01; ***P < 0.001; ****P < 0.0001; ns, not significant. Image acquisition, processing, and quantification were performed as in Figs. 3 to 5. Scale bars, 25 μm (whole-cell images) and 1 μm (insets).

According to our hypothesis, the accumulation of PIP_2_ at Gag assembly sites promotes the recruitment of CD43, PSGL-1, and CD44. To address this possibility, we measured the density of PIP_2_ spots found within a radius of 0.5 μm from a Gag spot in expanded cells (area A) and compared it with the density of PIP_2_ spots found within a radius of 2 μm excluding area A (area B) (Fig. 5C). This analysis showed that the PIP_2_ density in area A is 1.5 to 8 times higher than that found in area B with the average 4.6-fold enrichment of PIP_2_ in area A relative to area B (Fig. 5D). Notably, the presence of the Fyn(10) sequence on Gag does not affect PIP_2_ enrichment in the vicinity of Gag (compare fig. S7A with Fig. 5D). To test the role played by Gag MA-HBR in PIP_2_ clustering, we compared Fyn(10)/Gag-Venus with Fyn(10)/6A2T/Gag-Venus in which the basic residues in MA-HBR were substituted with neutral amino acids (Fig. 6, A and B, and fig. S7B). We observed that these substitutions led to a significant, albeit small, increase in the distance from PIP_2_ to Gag (Fig. 6, A to C, and fig. S7C) even when cells with similar numbers of cell surface Gag spots were compared (fig. S7, D and E). Consistent with this observation, basic-to-neutral substitutions in MA-HBR caused lower PIP_2_ enrichment in the vicinity of Gag (fig. S7F). Together, these results demonstrate that PIP_2_ is denser in the vicinity of Gag at the PM and are consistent with the hypothesis that MA-HBR interactions with PIP_2_ induce PIP_2_ accumulation at the virus assembly sites, which, in turn, promote the recruitment of CD43, PSGL-1, and CD44.

### PIP_2_ is enriched in the vicinity of CD43, PSGL-1, and CD44

To test whether the JMPBS in CD43, PSGL-1, and CD44 interacts with PIP_2_, cells were transfected with CD43 WT, CD43 6A, PSGL-1, CD44, or ICAM-1, probed for the respective protein and PIP_2_, and examined for the shortest distances between the transmembrane proteins and PIP_2_. The shortest distances from PIP_2_ to CD43 WT, PSGL-1, and CD44 were all significantly smaller than those from PIP_2_ to CD43 6A and ICAM-1 (Fig. 6, D to I). Next, we evaluated the capacity of these proteins to enrich PIP_2_ in their close proximity as was examined for Gag in Fig. 5 (C and D). CD43 WT, PSGL-1, and CD44 induced significantly higher enrichment of PIP_2_ in their proximity than CD43 6A and ICAM-1 (Fig. 6J). For CD43 WT, the PIP_2_ enrichment showed a relatively high correlation with the number of CD43 spots (*R*^2^ = ~0.62), suggesting that the abundance of the protein on the cell surface may partially contribute to the high PIP_2_ enrichment (fig. S8A). For PSGL-1 and CD44 (fig. S8, B and C), no correlation was observed (*R*^2^ = ~0.03 and ~0.02, respectively). These results indicate that CD43, PSGL-1, and CD44 have strong capacity to cause PIP_2_ enrichment in their proximity. In addition, this PIP_2_ accumulation seems to be JMPBS dependent at least for CD43, based on the differences observed between CD43 WT and 6A (Fig. 6J). In cells cotransfected with CD43 WT, Fyn(10)/Gag-Venus exhibited smaller shortest distances from PIP_2_ and higher PIP_2_ enrichment in its proximity than in cells transfected solely with Fyn(10)/Gag-Venus (see fig. S9, A and B), suggesting a possible synergy mechanism for PIP_2_ enrichment at viral assembly sites.

## DISCUSSION

PIP_2_ plays critical roles in various cellular functions as both a ligand for effector proteins and a signaling molecule (*14*). In HIV-1 assembly, PIP_2_ is known to recruit Gag to the PM as a membrane-associated ligand. Here, we demonstrated a hitherto unknown role for PIP_2_ wherein this lipid promotes recruitment of cellular transmembrane proteins CD43, PSGL-1, and CD44 into assembling HIV-1 particles. Cellular PIP_2_ depletion diminishes this incorporation without major effects on their trafficking to the PM or involvement of actin cytoskeleton (Fig. 1 and figs. S2 and S3). ExM showed that PIP_2_ facilitates coclustering between Gag and CD43, PSGL-1, or CD44 at the PM (Fig. 4) and that PIP_2_ accumulates in the vicinity of these proteins (Figs. 5 and 6). Although most PIP_2_ depletion experiments were performed using Fyn(10)/Gag constructs, which were necessary to circumvent the need for PIP_2_ in Gag PM binding, the degrees of coclustering with PSGL-1 or PIP_2_ are indistinguishable between Fyn(10)/Gag and natively N-myristoylated Gag (figs. S5, A to C, and S7A). Together, this study provides evidence for a PIP_2_-mediated mechanism for host protein sorting into HIV-1 assembly sites at the PM. As a number of enveloped viruses, including Ebola and influenza A viruses (*50*–*56*), rely on PIP_2_ for efficient assembly, it is conceivable that the PIP_2_-dependent mechanism observed here promotes the incorporation of host and/or viral transmembrane proteins into a broad range of viruses.

Previous lipidomic studies demonstrated the enrichment of PIP_2_ in HIV-1 particles compared to the PM (*31*, *32*). In addition, a microscopy-based study conducted in live cells revealed that Gag reduces motility of fluorescently labeled PIP_2_ when it is in close proximity to Gag (*30*), formally demonstrating Gag-PIP_2_ interactions in cells. Furthermore, at least in the in vitro studies, Gag has been shown to induce PIP_2_ clustering on the liposome (*57*, *58*). Consistent with these observations, the ExM analysis of the whole-cell PIP_2_ distribution in this study revealed the PIP_2_ enrichment in the vicinity of Gag. Although PIP_2_ enrichment in assembled HIV-1 particles has been recognized for many years, its functional significance in the fate of virus particles has remained elusive. Our study now reveals that PIP_2_ enrichment at virus assembly sites promotes the incorporation of the cellular transmembrane proteins that are known to modulate virus attachment to target cells, CD43, PSGL-1, and CD44 (*25*–*28*). Studies aimed at identifying the virus particle assembly step(s) at which the PIP_2_ enrichment and recruitment of the host transmembrane proteins take place are currently ongoing.

Basic-to-neutral mutations in the JMPBS of PSGL-1 and CD44 had mild and statistically nonsignificant impact, respectively, on their incorporation into HIV-1 particles compared to those in CD43 (Fig. 1). This observation implies the existence of additional mechanism(s) governing the sorting and incorporation of PSGL-1 and CD44 into HIV-1, mediated by other regions of the transmembrane proteins that interact with either PIP_2_ or other molecular partners. Consistent with this possibility, the deletion of the entire CT of PSGL-1 had a more pronounced impact on its coclustering with Gag than amino acid substitutions in its JMPBS (*23*). Furthermore, CD44 undergoes palmitoylation as a posttranslational modification, which can promote association to lipid rafts (*59*). Therefore, it is plausible that upon disruption of JMPBS, CD44 retains its ability to be incorporated into HIV-1 through the association with lipid raft(-like) microdomains that are also enriched at the assembly sites and can promote viral incorporation (*18*–*21*, *30*). We also do not rule out the possibility that other acidic phospholipid, such as phosphatidylserine, plays a role in the incorporation of CD43, PSGL-1, and CD44 into HIV-1. Whether and how additional mechanisms other than PIP_2_ coclustering promote sorting of CD43, PSGL-1, and CD44 to the virus assembly sites are subjects of further investigation.

Although ICAM-1 is known to be incorporated into HIV-1 particles (*36*, *60*), our previous study showed that replacing the CT with that of PSGL-1 significantly enhances the incorporation (*23*). Notably, ICAM-1 also has a polybasic sequence in the juxtamembrane region of the CT (*61*); however, our current study revealed that unlike CD43, PSGL-1, and CD44, ICAM-1 incorporation into VLPs and ICAM-1 distribution relative to Gag were insensitive to the presence or absence of PIP_2_ (Fig. 4 and fig. S1). Consistent with these results, ICAM-1 showed poor enrichment of PIP_2_ in its proximity compared to CD43, PSGL-1, and CD44 (Fig. 6). These results indicate that PIP_2_ plays an active role in the recruitment of CD43, PSGL-1, and CD44, but not ICAM-1, to HIV-1 assembly sites. Notably, ICAM-1 showed a moderate correlation between its abundance and its coclustering with Gag at the PM, in contrast to CD43, PSGL-1, and CD44, for which no correlations were observed between their respective abundances and Gag coclustering (fig. S6). Therefore, it is likely that the incorporation of ICAM-1 into HIV-1 particles is a passive event that depends on its PM abundance. It remains to be determined what prevents the polybasic sequence of ICAM-1 from recruiting PIP_2_ in the context of HIV-1 infection.

Multiple molecular dynamics simulation studies have shown that PIP_2_ enriched near the basic residues of various proteins, including CD44 (*59*, *62*–*64*), but cell-based evidence of PIP_2_ clustering in the proximity of a transmembrane protein has been limited thus far. A study on PM sheets prepared by cell sonication probed with a recombinant PIP_2_ biosensor or an anti-PIP_2_ antibody has shown PIP_2_ clustering in the vicinity of syntaxin-1A, a transmembrane protein with a JMPBS (*65*). Notably, biosensor-based analysis of PIP_2_ distribution tends to focus on the PIP_2_ population that is unengaged with cellular PIP_2_–interacting proteins and freely diffuses over the PM (*66*). Therefore, biosensor-based detection potentially underestimates PIP_2_ enrichment caused by interactions with basic residues of viral or cellular proteins. Our data obtained using an anti-PIP_2_ antibody showed that cellular proteins CD43, PSGL-1, and CD44, but not a CD43 variant lacking JMPBS, are associated with high density of native PIP_2_ in the intact cell context, providing additional evidence for transmembrane protein–induced PIP_2_ clustering in cells. Furthermore, these findings suggest a general mechanism by which transmembrane proteins with a JMPBS induce PIP_2_ clustering, which, in turn, contributes to coclustering with different proteins that have a PIP_2_-binding region, such as Gag (see below).

Although the ExM experiments revealed the enrichment of PIP_2_ within the distance of 0.5 μm from Gag, which corresponds to 100 to 120 nm in cells before expansion, this is unlikely to inform us on the actual size of PIP_2_ clusters. The expansion of cells four- to fivefold only achieves the resolution of approximately 60 to 80 nm depending on the fluorophore, precluding the measurement of a smaller PIP_2_ cluster size. In addition, the size of Gag-Venus and the extracellular domains of the cellular transmembrane proteins as well as primary and secondary antibodies for detecting them, which are all >10 nm, introduces uncertainty to distance measurement. Nonetheless, considering that Gag coclustering with PSGL-1 occurs within the range of up to 200 nm (*23*), it appears possible that the formation of the PIP_2_-enriched areas in the vicinity of Gag depends not only on direct short-range Gag-PIP_2_ interactions but also on the formation of electrostatic network mediated by other positively charged molecules such as divalent cations.

The magnitudes of PIP_2_ enrichment caused by the cellular transmembrane proteins appear larger than that observed with Gag. However, because the ways that Gag and the cellular transmembrane proteins studied here bind to PIP_2_ are different (*11*–*13*, *62*, *67*–*69*), which could affect the efficiencies of PIP_2_ headgroup detection by the anti-PIP_2_ antibody, it is not possible to compare the capacity to induce PIP_2_ clustering between the cellular transmembrane proteins and Gag. Nonetheless, our data showing the robust PIP_2_ enrichment in the vicinity of the cellular transmembrane proteins suggest interesting possibilities that HIV-1 Gag can be targeted to PIP_2_-enriched areas induced by the cellular transmembrane proteins and that recruiting the cellular transmembrane proteins expands PIP_2_ clusters at the assembly sites. In support of the latter possibility, we observed higher PIP_2_ enrichment in the proximity of Gag in the presence of CD43 than in its absence (fig. S9). Notably, an in vitro study demonstrated that the myristoylated MA of Gag prefers to bind clustered rather than free PIP_2_ in liposomes (*57*). Therefore, it is tempting to speculate that HIV-1 exploits PIP_2_ clusters made by cellular transmembrane proteins to facilitate Gag recruitment to assembly sites.

In summary, we demonstrate that Gag and the cellular transmembrane proteins CD43, PSGL-1, and CD44 induce local PIP_2_ enrichment and that PIP_2_ is essential for the sorting and incorporation of these proteins into HIV-1 particles. Together, PIP_2_ at virus assembly sites, by bridging the association of CD43, PSGL-1, and CD44 with Gag, emerges as a key player in shaping the unique composition of the viral envelope, ultimately modulating viral spread.

## MATERIALS AND METHODS

### Cells and plasmids

HeLa cells were cultured and transfected as previously described (*23*). The plasmids used in transfection for the expression of HIV-1 Gag proteins were pNL4-3/Gag-Venus (*15*, *16*), pNL4-3/1GA/6A2T/Gag-Venus, pNL4-3/Fyn(10)/Gag, pNL4-3/Fyn(10)/Gag-Venus, and pNL4-3/Fyn(10)/6A2T/Gag-Venus (*16*). These plasmids are HIV-1 molecular clones and express HIV-1 Tat. For the expression of uropod proteins, the following plasmids were used for transfection: pCMV6-AC/CD43/WT or 6A, pCMV6-AC/PSGL-1/WT or 3A, pCMV6-AC/PSGL-1/ΔCT, pCMV6-AC/CD44 or 6A, pCMV6-AC/ICAM-1, and pCMV6-AC/Empty-Vector (*23*). For PIP_2_ depletion experiments, pHIV–Myc–5ptaseIV FL, which expresses 5ptaseIV in a Tat-dependent manner, was used (*13*). As a negative control, pHIV–Myc–5ptaseIV Δ1, which contains a deletion encompassing the enzyme active site, was used instead (*13*). For the analysis of CD44-Gag coclustering and CD44 incorporation into virions, we used HeLa cells that are depleted of endogenous CD44 (CD44 KO) using the CRISPR-Cas9 approach (*28*). These cells are also referred to as HeLa cells in Results.

### Antibodies

The antibodies against CD43 (1G10), PSGL-1 (clone KPL-1), CD44 (515), and ICAM-1 (LB-2) were obtained from BD Pharmingen. The antibodies against CD44 (2C5) and ICAM-1 (EP1442Y) for Western blotting analysis were obtained from R&D Systems and Abcam, respectively. Anti-HIV immunoglobulin (Ig) was obtained from National Institutes of Health AIDS Research and Reference Reagent Program. HIV-1 core antigen–fluorescein isothiocyanate (FITC; KC57) was obtained from Beckman Coulter. For the detection of Fyn(10)/Gag-Venus in ExM, we used a rabbit anti–green fluorescent protein (GFP) (Sigma-Aldrich, SAB4701015). Free PIP_2_ was detected using mouse anti-PI(4,5)P_2_ (Echelon Biosciences, Z-PD45). For flow cytometry experiments, the primary antibodies were conjugated with Alexa Fluor 647 following the manufacturer’s protocol (Invitrogen Antibody Labeling Kit, A20186). Secondary antibodies for immunofluorescence used were Invitrogen Alexa Fluor 488 goat anti-rabbit (A11008), Alexa Fluor 594 goat anti-mouse IgM μ chain (A21044), Alexa Fluor 488 goat anti-mouse (A11001), and Alexa Fluor 594 goat anti-mouse (11005).

### Viral incorporation assays

The viral incorporation assays were performed as previously described with modifications (*25*). Briefly, either 350,000 CD44 KO or WT HeLa cells were seeded onto six-well plates and maintained in 5% Dulbecco’s modified Eagle’s medium without penicillin and streptomycin. On the next day, the cells were transfected with 3 μg of pNL4-3/Fyn(10)/Gag, 0.85 μg of plasmids encoding the cellular uropod proteins, and 1 μg of pHIV–Myc–5ptaseIV FL or Δ1 using Lipofectamine 2000. At 16 to 18 hours post-transfection, the supernatants were collected, passed through a 0.45-μm filter, and ultracentrifuged at 35,000 rpm for 95 min at 4°C. In actin disruption experiments, at 12 hours post-transfection, the medium was removed, and a fresh medium containing dimethyl sulfoxide (control) or 10 μM Lat B was added. Four hours later, the viral and cell pellets were suspended in Triton X-100 lysis buffer [0.5% Triton X-100, 300 mM NaCl, and 50 mM tris-HCl (pH 7.5)] containing protease inhibitors (cOmplete, MilliporeSigma, 11836170001). The cell and virus lysates were resolved using a discontinuous 6 to 10% (for fig. S2) or 8 to 10% (for Fig. 1) SDS-polyacrylamide gel, followed by transfer to a polyvinylidene difluoride membrane. For the detection by immunoblotting, the membranes were blocked in SuperBlock (Thermo Fisher Scientific, PI37515) solution and probed using the primary antibodies (see the “Antibodies” section) indicated in each corresponding figure. The chemiluminescent signal was detected using either West Pico or West Femto chemiluminescence substrate (Thermo Fisher Scientific, PI34580 and PI34696, respectively) and recorded with a GeneSys image acquisition system (Syngene). The viral incorporation of CD43, PSGL-1, CD44, and ICAM-1 was calculated as follows: The intensity of the cellular transmembrane protein bands in the viral supernatant was normalized first by the intensity of their corresponding bands in the cell lysates and then by the HIV p24 levels in the viral lysates.

### Expansion microscopy

The ExM experiments were performed as described previously (*47*) with some modifications. Briefly, HeLa cells were seeded onto 12-mm coverslips and transfected as described above, except that the ratio of the plasmids encoding Fyn(10)/Gag-Venus and 5ptaseIV FL or Δ1 was 1:1. At 16 to 18 hours post-transfection, the cells were fixed in phosphate-buffered saline (PBS) containing 4% paraformaldehyde (PFA) and 0.2% glutaraldehyde for 30 min. For detecting only PM population of the host transmembrane proteins, the cells were rinsed five times in PBS and probed with appropriate primary antibody for 1 hour and rinsed at least 10 times in PBS. VenusYFP-tagged Gag derivatives were detected using anti-GFP following permeabilization of cells with 0.1% saponin. For detecting PI(4,5)P_2_, we adapted a previously described method (*49*). First, the cells were permeabilized for 45 min in 0.3% saponin solution, followed by the anti-PIP_2_ antibody incubation for 1 hour. Both processes were done on ice. The cells incubated with primary antibodies were rinsed 10 times in cold PBS and incubated with solutions containing secondary antibodies for 1 hour. The cells were rinsed at least 10 times in cold PBS and fixed again with 2% PFA solution. The cells were rinsed 10 times in PBS at room temperature, and then the cells were incubated in PBS containing 0.54% of acrylamide and 0.33% PFA (the hybridization solution) overnight at 37°C. Subsequently, the cells were washed three times for 10 min each and incubated in the hydrogel solution containing 19% of sodium acrylate (Sigma-Aldrich, 408220; Pfaltz & Bauer, SO3880), 10% acrylamide (Sigma-Aldrich, A9099), 0.1% *N*,*N*′-(di-hydroxy-ethylene bis-acrylamide) (DHEBA; Sigma-Aldrich, 294381), 0.25% ammonium persulfate (APS), and 0.25% N,N,N',N'-tetramethylethylenediamine (TEMED). The coverslips were incubated for 15 min at room temperature and then for 2 hours in a humidified chamber at 37°C. After that, the cell-containing gels were carefully detached from the coverslips using a spatula, incubated in a denaturation buffer [200 mM SDS, 200 mM NaCl, and 40 mM tris-HCl (pH 6.8)] at room temperature for 15 min, transferred to a 1.5-ml tube containing 1 ml of the fresh denaturation buffer, and further incubated at 63°C for 1 hour. The gels were placed in petri dishes, washed in 30 ml of Milli-Q water at least two times, 1 hour each, and then incubated in fresh Milli-Q water overnight. On the next day, the water was removed, and the gels were incubated in a 30% glycerol (w/v in water) solution overnight. Pieces of the gels were cut off, the excess water was carefully removed from them, and the gel pieces were placed on coverslips pretreated with poly-l-lysine (0.1% w/v in water). The gels were imaged using a Nikon Ti2 coupled with Yokogawa Spinning Disk Microscope, using 405-, 488-, and 594-nm excitation lasers. The objective used was 100× oil with a numerical aperture of 1.4. The *z*-stack images taken were reconstructed in Imaris 10.0.1 software (Oxford), with which the quantitative analyses were also performed.

### Flow cytometry

At 16 hours post-transfection, the transfected cells were rinsed once with PBS and detached with 2 mM EDTA in PBS for 1 min. The cells were pelleted down and resuspended in PBS containing 4% PFA and 0.1% glutaraldehyde. For the analysis of the cellular transmembrane protein PM expression levels, the cells were fixed, washed, and probed with mouse anti-CD43, anti–PSGL-1, or anti-CD44, which are directly conjugated with Alexa Fluor 647 (see the “Antibodies” section for more details) for 1 hour at 37°C. For evaluating the expression levels of the cellular transmembrane proteins in a whole cell (PM + cytoplasmic), the cells were fixed, washed, permeabilized with PBS containing 0.2% Triton X-100 for 5 min at room temperature, and then probed with the same antibodies described above. Then, the cells were incubated with FITC-conjugated anti–HIV-1 p24 (clone KC57) for 1 hour, washed again with 3% bovine serum albumin in PBS, and analyzed using a BD LSR Fortessa flow cytometer. The data acquired were analyzed in a FlowJo software. Cells transfected with pUC19 and pCMV6-AC/Empty were used to set the gates for expression of Gag and cellular transmembrane proteins, respectively. The mean fluorescence intensity was determined for the cells positive for both Gag and cellular transmembrane proteins.

### Cell viability

HeLa cells were transfected with pNL4-3/Fyn(10)/Gag-Venus along with 5ptaseIV ∆1 or FL. As controls, the HeLa cells were treated with Lipofectamine only or cotransfected with pNL4-3/Fyn(10)/Gag-Venus alone. At 16 to 18 hours post-transfection, the cells were detached with 2 mM EDTA for 1 min. The cells were pelleted and incubated with Zombie Dye NIR reagent (BioLegend 423105) according to the manufacturer’s protocol. The cells were then fixed and analyzed by flow cytometry.

### Data analysis

Fiji ImageJ software (W. S. Rasband, ImageJ, U.S. National Institutes of Health, Bethesda, MD, USA, https://imagej.net/ij/, 1997–2018) was used to display and analyze immunoblots and to display expanded and non-expanded fluorescence images. All plots were prepared using GraphPad Prism version 9.0. ExM images were analyzed using Imaris software version 9.91 and 10.0.1. Fluorescent signals were segmented into Imaris spots using Imaris spots creation wizard. The shortest centroid-to-centroid distances between all Gag spots and spots representing other labels were measured using the filter parameter Shortest distance, and the average shortest distance for all Gag spots in individual cells is reported within violin plots. 3D reconstructions were generated using Spots Growing Region and Background subtraction algorithms using 0.35 μm for diameter. Randomized distributions were generated in Matlab by first estimating a tessellated cell surface from spot centroids using the alphashape function, and then points were redistributed randomly on this surface using the randtess function. Nearest neighbor distances were then tabulated from randomized points.

### Statistical analysis

Statistical analyses were performed using Prism 10 (GraphPad Software Inc., USA). The *P* value was determined using nonpaired or paired Student’s *t* test or analysis of variance (ANOVA) one-way Tukey’s multiple-comparison test as described in figure legends.
