# Supplementary material for: PIP2 promotes the incorporation of CD43, PSGL-1, and CD44 into nascent HIV-1 particles
Source: Sci Adv. 2025 Apr 4;11(14):eads9711. doi: 10.1126/sciadv.ads9711 (PMC11970457; doi:10.1126/sciadv.ads9711)
Supplement: Supplementary file 1 — Figs. S1 to S9 [file sciadv.ads9711_sm.pdf]

Science Advances
AAAS

Supplementary Materials for

PIP$_2$ promotes the incorporation of CD43, PSGL-1, and CD44 into nascent HIV-1 particles

Ricardo de Souza Cardoso *et al.*

Corresponding author: Akira Ono, akiraono@umich.edu

Sci. Adv. **11**, eads9711 (2025)
DOI: 10.1126/sciadv.ads9711

This PDF file includes:

Figs. S1 to S9

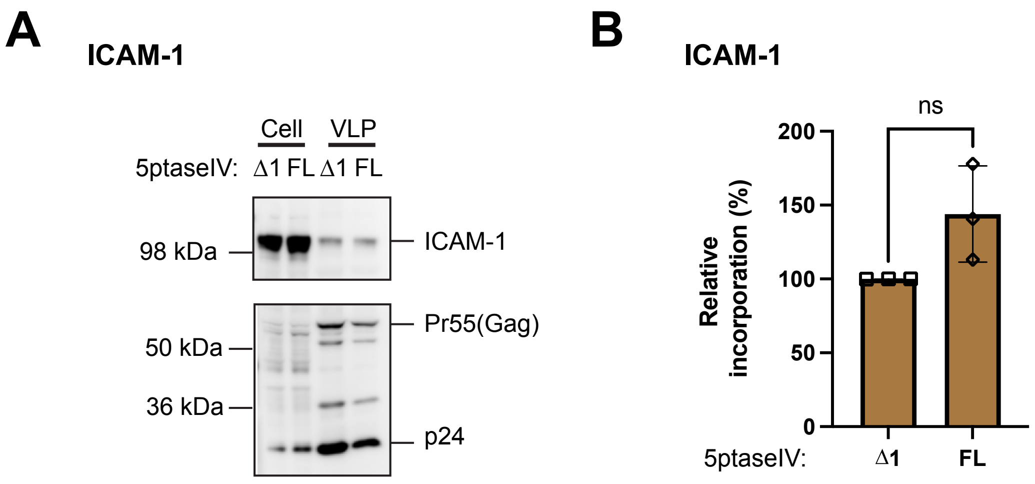
A
ICAM-1
Cell
VLP
5ptaseIV: Δ1 FL Δ1 FL
98 kDa
ICAM-1
Pr55(Gag)
50 kDa
36 kDa
p24
B
ICAM-1
ns
Relative incorporation (%)
200
150
100
50
0
5ptaseIV:
Δ1
FL

Fig. S1.

The effect of PIP2 depletion on ICAM-1 incorporation into VLPs. HeLa cells were transfected with plasmids encoding Fyn(10)/Gag, ICAM-1, and 5ptaseIV Δ1 or Full Length (FL). Western blotting analysis of cell and viral lysates were performed for ICAM-1 (**A**). In **B,** the incorporation efficiency was calculated as the ratio of ICAM-1 in viral lysates versus cell lysates, which was normalized for the amount of released particles represented by p24 in virus lysates. The relative incorporation efficiency for each condition was calculated in comparison to the incorporation efficiency of ICAM-1 into virus in the presence of 5ptaseIV Δ1. The data from three independent experiments are shown. The *P* values were determined by non-paired Student's *t* test (panel **B**). ns, nonsignificant.

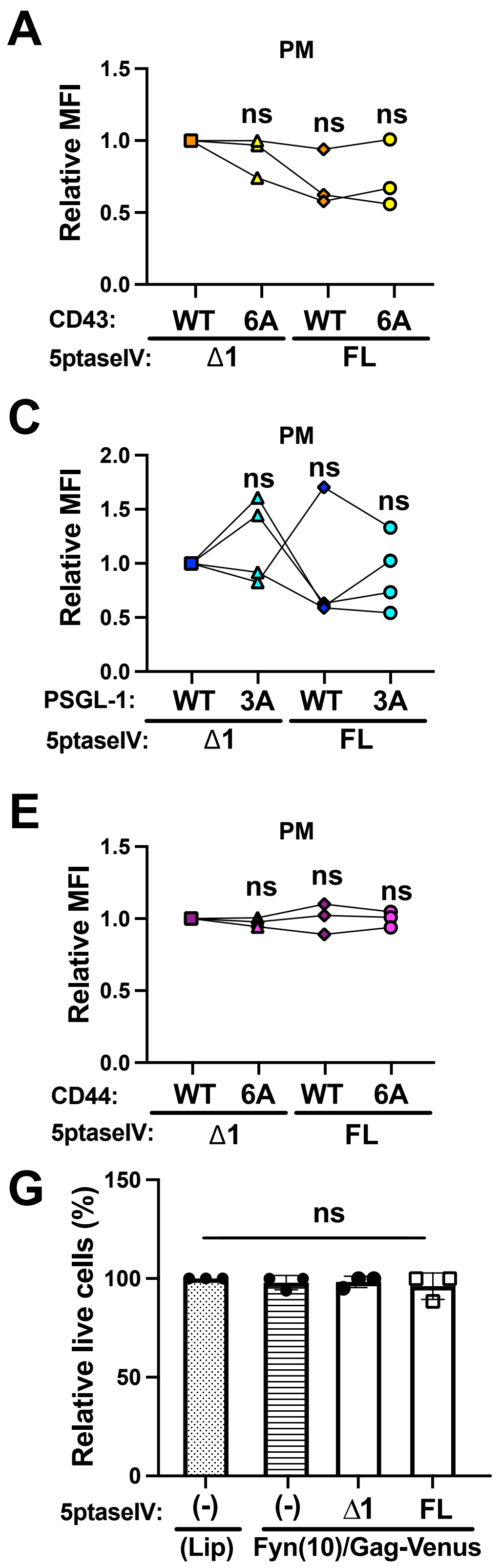

A
PM
Relative MFI
1.5
1.0
0.5
0.0
ns
ns
ns
CD43:
WT
6A
WT
6A
5ptaseIV:
Δ1
FL
B
PM + Cytoplasmic
Relative MFI
1.5
1.0
0.5
0.0
ns
ns
ns
CD43:
WT
6A
WT
6A
5ptaseIV:
Δ1
FL
C
PM
Relative MFI
2.0
1.5
1.0
0.5
0.0
ns
ns
ns
PSGL-1:
WT
3A
WT
3A
5ptaseIV:
Δ1
FL
D
PM + Cytoplasmic
Relative MFI
2.0
1.5
1.0
0.5
0.0
ns
ns
ns
PSGL-1:
WT
3A
WT
3A
5ptaseIV:
Δ1
FL
E
PM
Relative MFI
1.5
1.0
0.5
0.0
ns
ns
ns
CD44:
WT
6A
WT
6A
5ptaseIV:
Δ1
FL
F
PM + Cytoplasmic
Relative MFI
1.5
1.0
0.5
0.0
ns
ns
ns
CD44:
WT
6A
WT
6A
5ptaseIV:
Δ1
FL
G
Relative live cells (%)
150
100
50
0
ns
5ptaseIV:
(-)
(-)
Δ1
FL
(Lip)
Fyn(10)/Gag-Venus

Fig. S2.

The expression levels of CD43, PSGL-1, and CD44 and their mutants at the plasma membrane and the effect of 5ptaseIV on cellular viability. A, C, and **E)** Mean fluorescence intensity of cellular transmembrane proteins was determined by flow cytometry for HeLa cells co-transfected with plasmids for CD43 WT or 6A (A and B), PSGL-1 WT or 3A (C and D), or CD44 WT or 6A (E and F) along with those for Fyn(10)/Gag-Venus and 5ptaseIV Δ1 or FL. PM, plasma membrane levels determined by analyzing cells immunostained without prior cell permeabilization in p24 positive populations; **B, D** and **F**) PM + Cytoplasmic, total protein expression levels determined by analyzing permeabilized cells in p24 positive populations. **G)** Cell viability assay in cells that were treated with Lipofectamine only (Lip) or cells transfected with Fyn(10)/Gag-Venus along with no plasmid (-) or plasmids for 5ptaseIV Δ1 or FL. The experiments were repeated three times. The *P* values were determined using analysis of variance (ANOVA) one-way Tukey's multiple-comparison test. ns, nonsignificant.

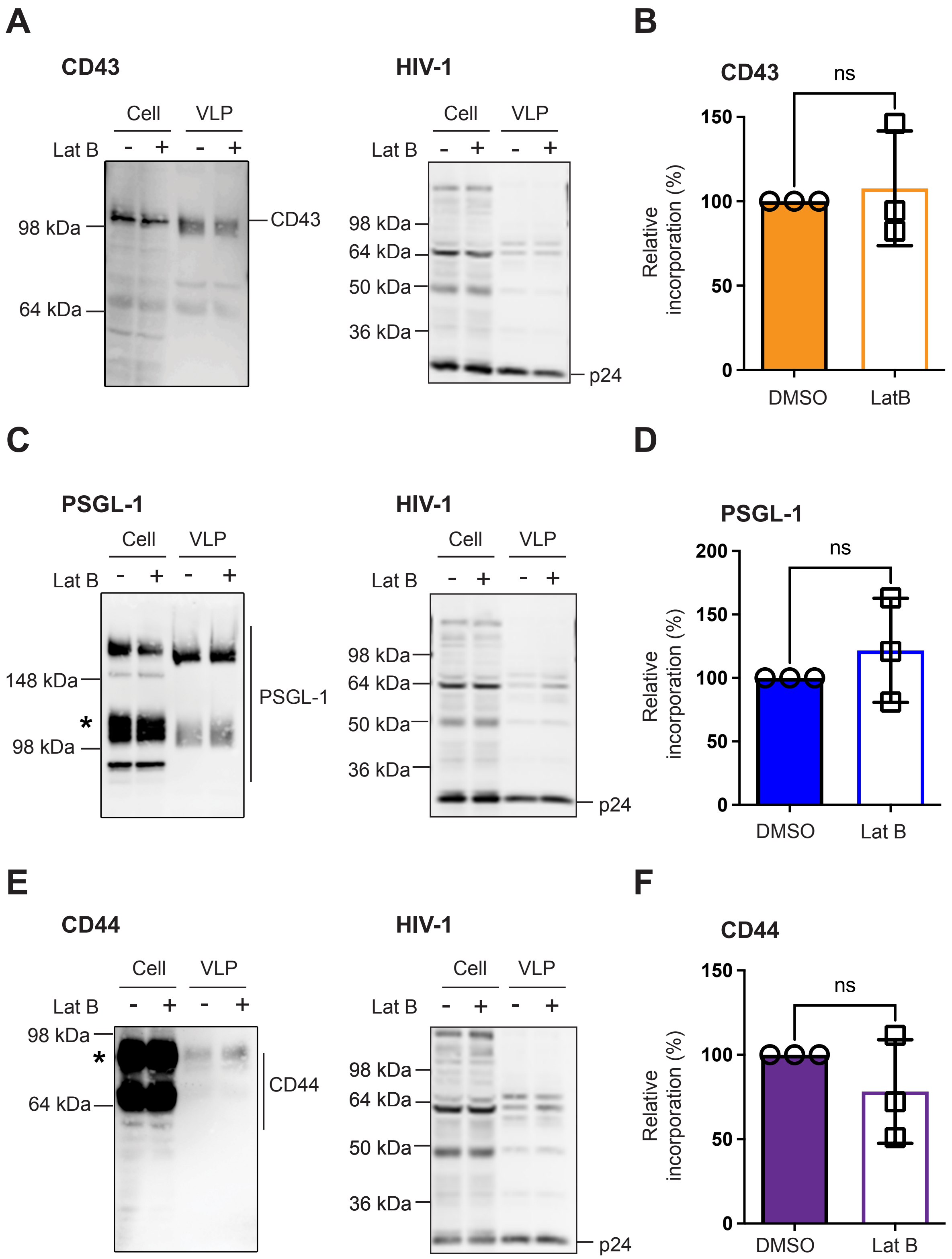
A
CD43
Cell
VLP
Lat B
-
+
-
+
98 kDa
64 kDa
CD43
HIV-1
Cell
VLP
Lat B
98 kDa
64 kDa
50 kDa
36 kDa
p24
B
CD43
ns
Relative incorporation (%)
150
100
50
0
DMSO
LatB
C
PSGL-1
Cell
VLP
Lat B
148 kDa
*
98 kDa
PSGL-1
HIV-1
Cell
VLP
Lat B
98 kDa
64 kDa
50 kDa
36 kDa
p24
D
PSGL-1
ns
Relative incorporation (%)
200
150
100
50
0
DMSO
Lat B
E
CD44
Cell
VLP
Lat B
98 kDa
*
64 kDa
CD44
HIV-1
Cell
VLP
Lat B
98 kDa
64 kDa
50 kDa
36 kDa
p24
F
CD44
ns
Relative incorporation (%)
150
100
50
0
DMSO
Lat B

Fig. S3.

The effect of actin disruption on CD43, PSGL-1 and CD44 incorporation into VLPs. A-F), HeLa cells expressing CD43 (**A** and **B**), PSGL-1 (**C** and **D**), or CD44 (**E** and **F**) along with HIV-1 encoding Fyn(10)/Gag was cultured in the presence of Latrunculin B or vehicle control DMSO for 4 hours. At 16 hours post transfection, cell and viral lysates were prepared and analyzed by western blotting analysis using antibodies against CD43, PSGL-1 and CD44 and HIV-Ig, and relative viral incorporation of the indicated proteins (**A**, **C**, and **E**) was determined as in Figure 1. The asterisks in Panels C and E denote the bands for PSGL-1 and CD44 quantitated for Panels D and F, respectively. The experiments were repeated three times. The *P* values were determined non-paired Student's *t* test (panels **B**, **D** and **F**). ns, nonsignificant.

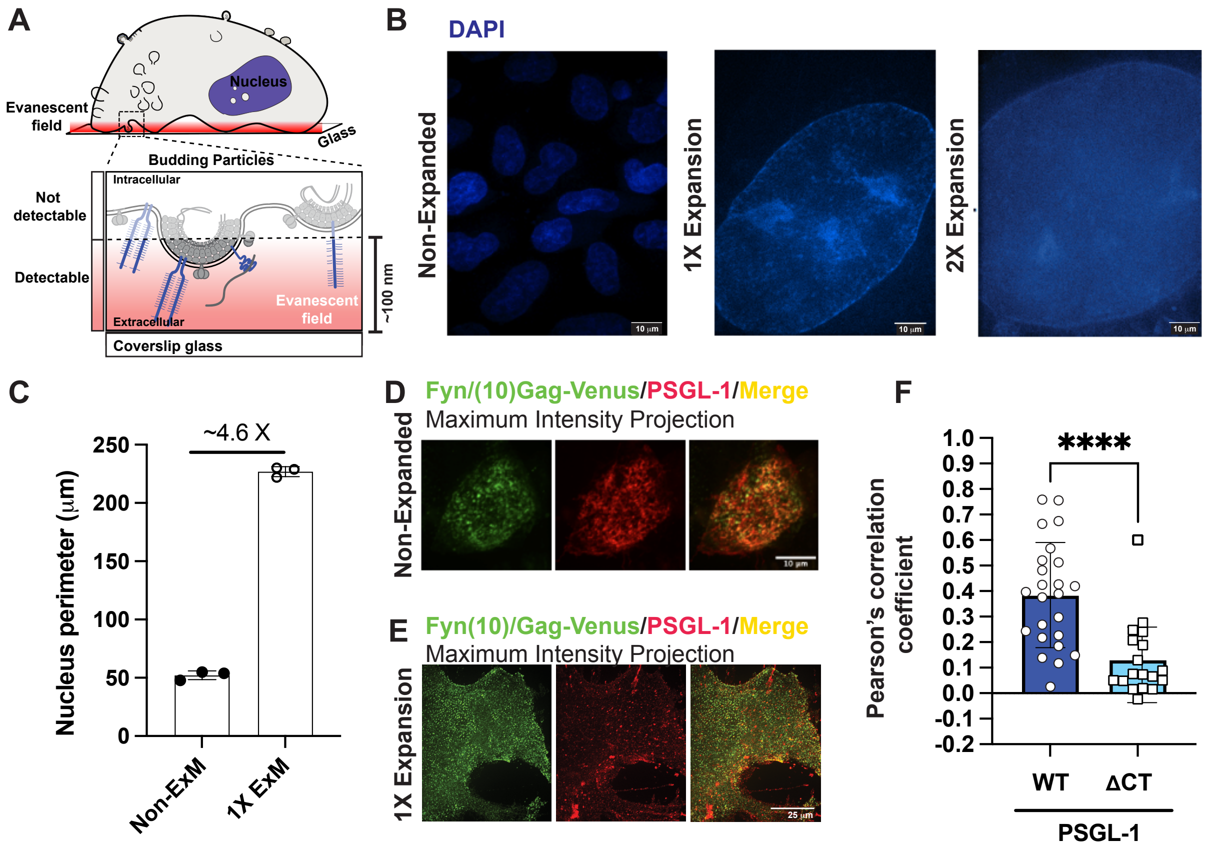

A
Nucleus
Evanescent field
Glass
Budding Particles
Intracellular
Not detectable
Detectable
Extracellular
Evanescent field
~100 nm
Coverslip glass
B
DAPI
Non-Expanded
1X Expansion
2X Expansion
10 μm
C
~4.6 X
Nucleus perimeter (μm)
250
200
150
100
50
0
Non-ExM
1X ExM
D
Fyn/(10)Gag-Venus/PSGL-1/Merge
Maximum Intensity Projection
Non-Expanded
E
Fyn(10)/Gag-Venus/PSGL-1/Merge
Maximum Intensity Projection
1X Expansion
25 μm
F
****
Pearson's correlation coefficient
1.0
0.9
0.8
0.7
0.6
0.5
0.4
0.3
0.2
0.1
0.0
-0.1
-0.2
WT
ΔCT
PSGL-1

Fig. S4.

Expansion microscopy standardization. A) Schematic representation of the potential caveat with TIRF-based super-resolution microscopy. Association of tall transmembrane proteins (shown in blue) with the assembling particles at the PM can be underestimated due to the limited depth of the detectable range. **B**) Representative images of nuclei in non-expanded HeLa cells or HeLa cells after one round or 2 rounds of expansion. **C**) Measurement of the expansion factor after one round of expansion. **D**-**E**) Maximum intensity projection of non-expanded or expanded HeLa cells co-transfected with pNL4-3/Fyn(10)/Gag-Venus and a plasmid encoding PSGL-1. **F**) Pearson's correlation coefficient analysis of cells expressing Fyn(10)/Gag-Venus with PSGL-1 WT or ΔCT analyzed in Fig. 3A-C. All the experiments were repeated at least three times, and at least eight cells from each biological replicate were analyzed. The *P* value was determined using non-paired analysis of Student's *t* test. ****, $P < 0.0001$. Image acquisition, processing, and quantification were performed as in Figure 2. Scale bars, 25 μm for **E** and 10 μm for **B** and **D**.

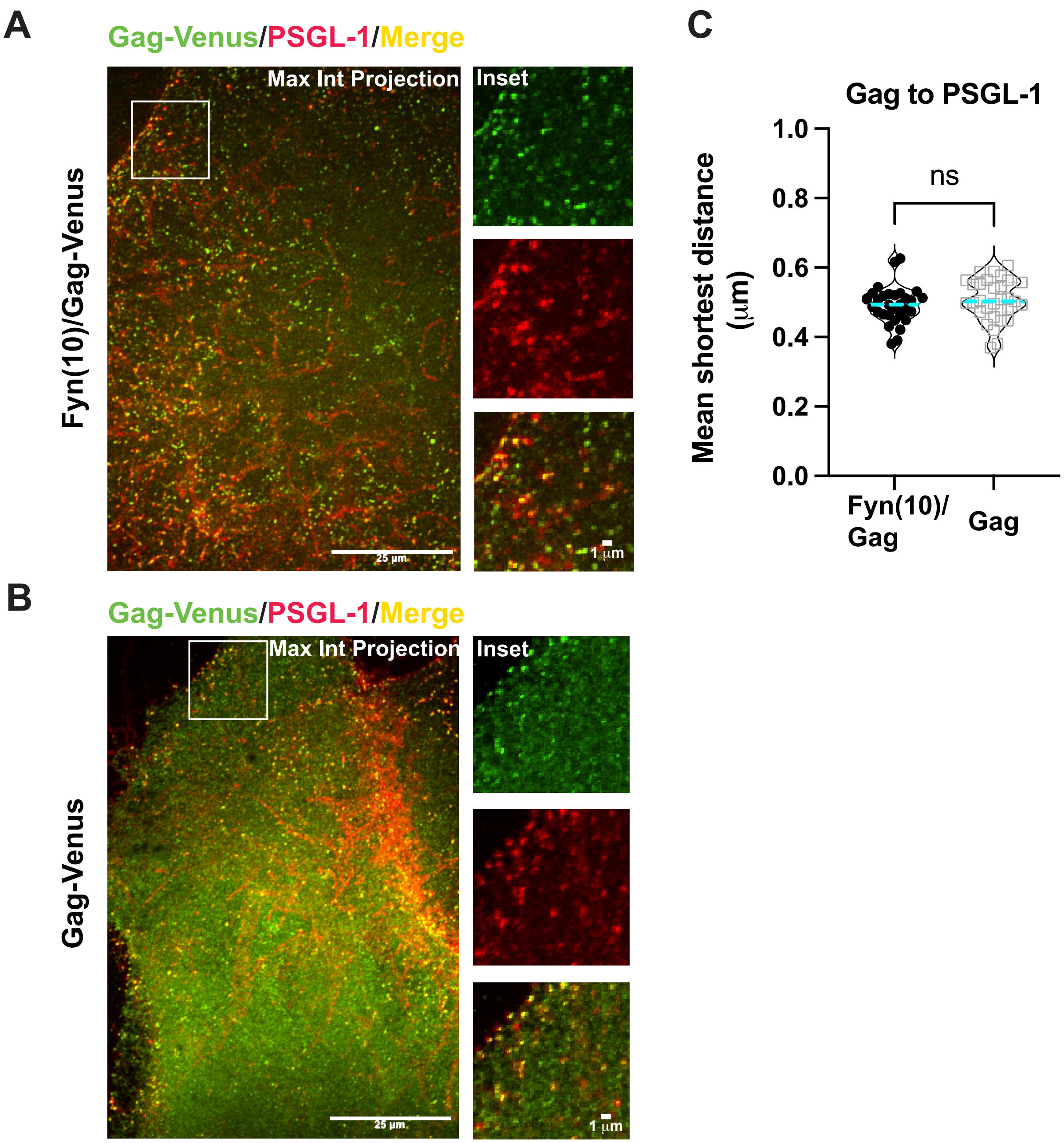
A
Gag-Venus/PSGL-1/Merge
Max Int Projection
Inset
Fyn(10)/Gag-Venus
25 µm
1 µm
B
Gag-Venus/PSGL-1/Merge
Max Int Projection
Inset
Gag-Venus
25 µm
1 µm
C
Gag to PSGL-1
Mean shortest distance (µm)
1.0
0.8
0.6
0.4
0.2
0.0
ns
Fyn(10)/ Gag
Gag

Fig. S5.

Comparison between Fyn(10)/Gag-Venus and Gag-Venus for coclustering with PSGL-1. **A** and **B**) Maximum intensity projections of cells transfected either with Fyn(10)/Gag-Venus or Gag-Venus along with PSGL-1 WT. The insets correspond to the boxed areas shown in whole cell images. **C)** Mean shortest distances from Fyn(10)/Gag-Venus or Gag-Venus to PSGL-1. All the experiments were repeated at least three times, and at least 10 cells from each biological replicate were analyzed The *P* value was determined using non-paired Student's *t* test analysis. ns, non-significant. Image acquisition, processing, and quantification were performed as in Figure 2. Scale bars, 25 μm for whole cell images and 1 μm for insets.

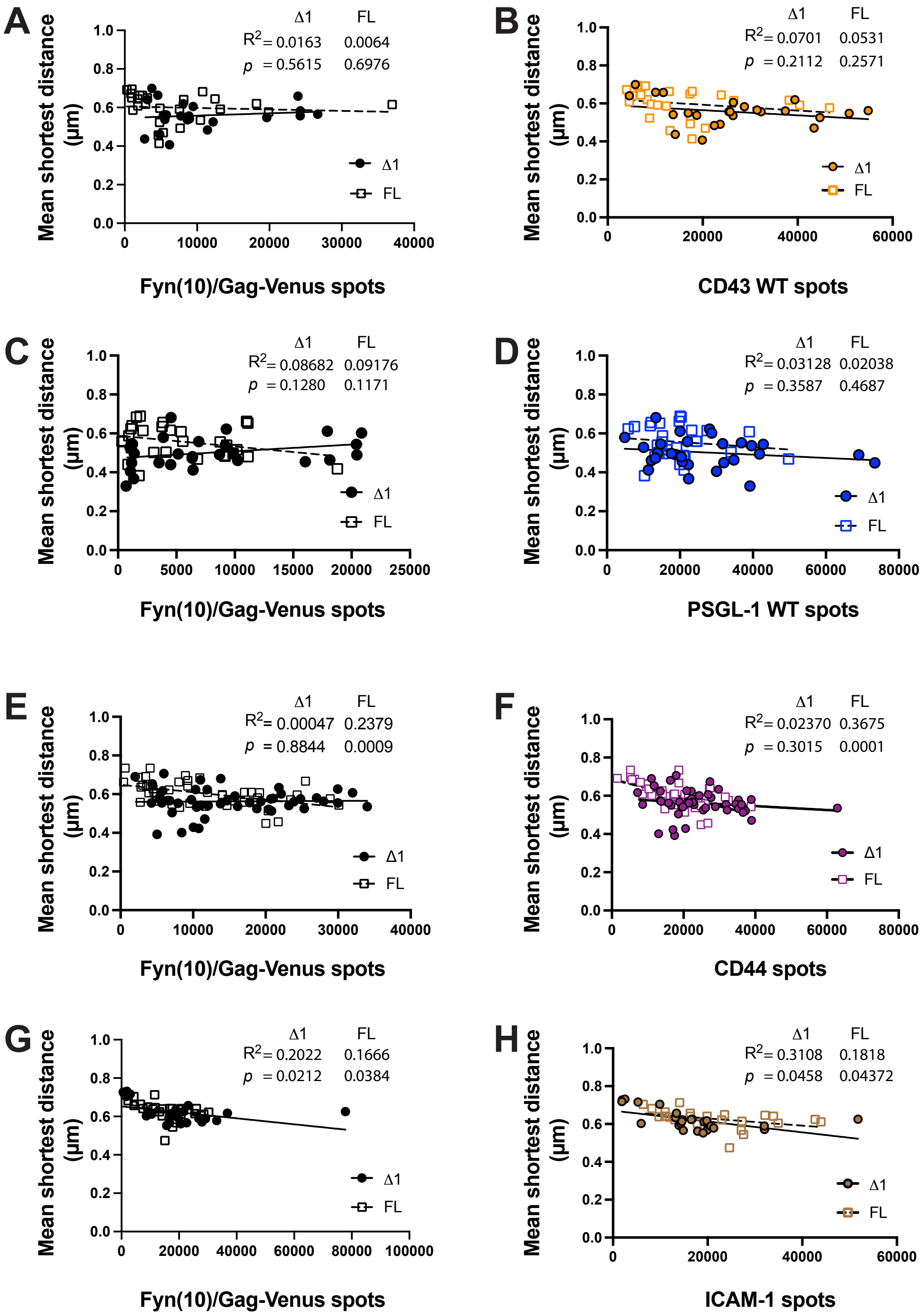

A
Mean shortest distance (μm)
Fyn(10)/Gag-Venus spots
Δ1 FL
R^2= 0.0163 0.0064
p = 0.5615 0.6976
Δ1
FL
B
Mean shortest distance (μm)
CD43 WT spots
Δ1 FL
R^2= 0.0701 0.0531
p = 0.2112 0.2571
Δ1
FL
C
Mean shortest distance (μm)
Fyn(10)/Gag-Venus spots
Δ1 FL
R^2= 0.08682 0.09176
p = 0.1280 0.1171
Δ1
FL
D
Mean shortest distance (μm)
PSGL-1 WT spots
Δ1 FL
R^2= 0.03128 0.02038
p = 0.3587 0.4687
Δ1
FL
E
Mean shortest distance (μm)
Fyn(10)/Gag-Venus spots
Δ1 FL
R^2= 0.00047 0.2379
p = 0.8844 0.0009
Δ1
FL
F
Mean shortest distance (μm)
CD44 spots
Δ1 FL
R^2= 0.02370 0.3675
p = 0.3015 0.0001
Δ1
FL
G
Mean shortest distance (μm)
Fyn(10)/Gag-Venus spots
Δ1 FL
R^2= 0.2022 0.1666
p = 0.0212 0.0384
Δ1
FL
H
Mean shortest distance (μm)
ICAM-1 spots
Δ1 FL
R^2= 0.3108 0.1818
p = 0.0458 0.04372
Δ1
FL

Fig. S6.

Analysis of correlation between shortest distance and plasma membrane signal abundance. **A**, **C**, **E**, and **G**) Correlation between the number of Fyn(10)/Gag-Venus spots and the mean shortest distance from Fyn(10)/Gag-Venus to CD43 (A), PSGL-1 (C), CD44 (E), or ICAM-1 (G). **B**, **D**, **F**, and **H**) Correlation between the number of CD43 (B), PSGL-1 (D), CD44 (F), and ICAM-1 (H) spots and the mean shortest distance from the indicated transmembrane proteins to Fyn(10)/Gag-Venus. Each dot represents a cell examined in Figure 4 for the shortest distance between fluorescent spots representing the indicated transmembrane proteins and Fyn(10)/Gag-Venus. The experiments were repeated at least three times. The *P* values and the R^2 are annotated in each corresponding graph.

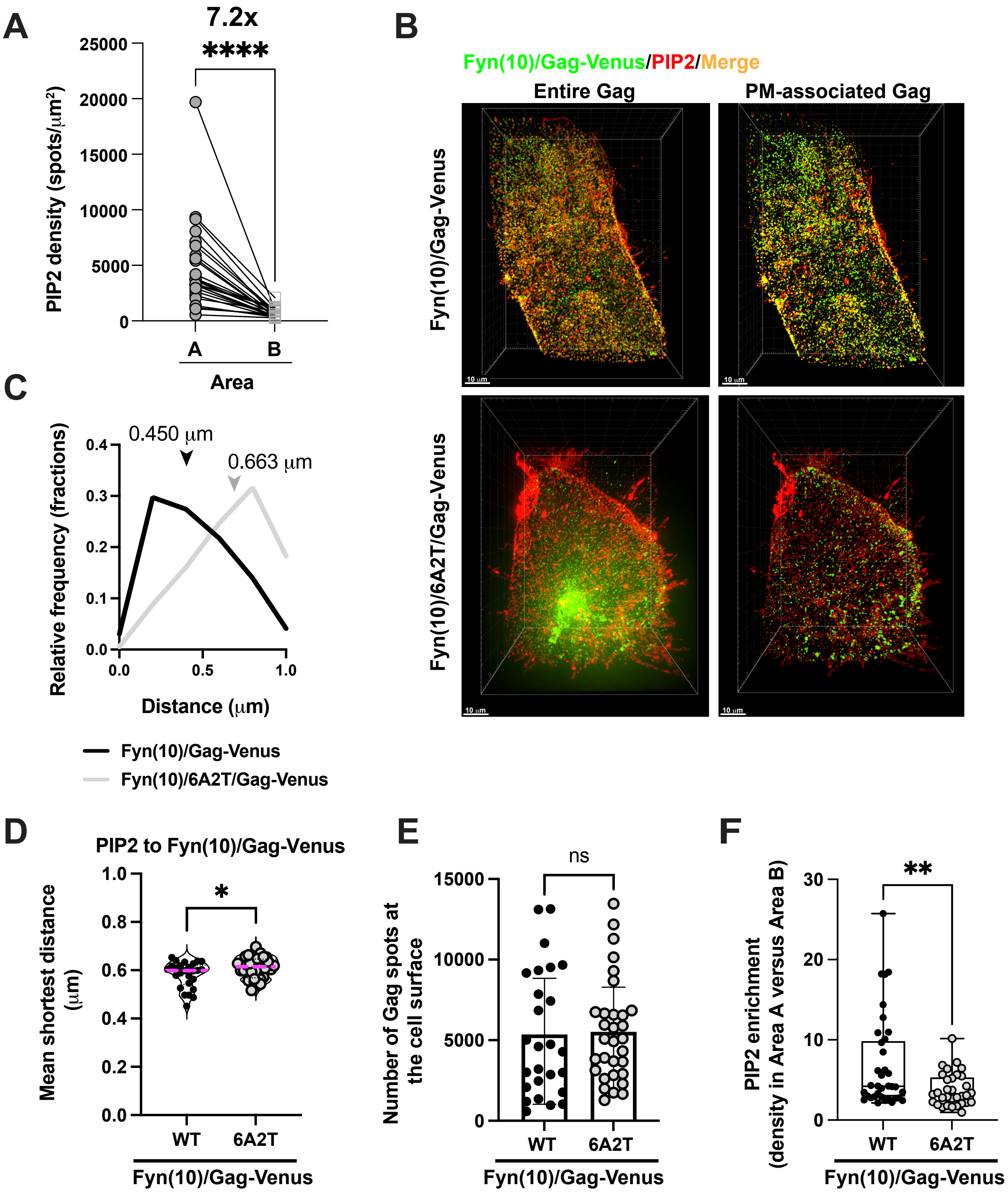
A
7.2x
****
PIP2 density (spots/μm²)
25000
20000
15000
10000
5000
0
A
B
Area
B
Fyn(10)/Gag-Venus/PIP2/Merge
Entire Gag
PM-associated Gag
Fyn(10)/Gag-Venus
Fyn(10)/6A2T/Gag-Venus
C
0.450 μm
0.663 μm
Relative frequency (fractions)
0.4
0.3
0.2
0.1
0.0
0.0
0.5
1.0
Distance (μm)
Fyn(10)/Gag-Venus
Fyn(10)/6A2T/Gag-Venus
D
PIP2 to Fyn(10)/Gag-Venus
Mean shortest distance (μm)
1.0
0.8
0.6
0.4
0.2
0.0
*
WT
6A2T
Fyn(10)/Gag-Venus
E
ns
Number of Gag spots at the cell surface
15000
10000
5000
0
WT
6A2T
Fyn(10)/Gag-Venus
F
**
PIP2 enrichment (density in Area A versus Area B)
30
20
10
0
WT
6A2T
Fyn(10)/Gag-Venus

Fig. S7.

The effect of the MA highly basic region on Gag-PIP2 co-clustering. A) PIP2 density in the Areas A (within a radius of 0.5 µm from a Gag-Venus spot) and B (within a radius of 2 µm from Gag-Venus but excluding area A) was measured as the number of PIP2 spots normalized for the area size as in Figure 5D. Note that PIP2 enrichment is observed in the vicinity of Gag-Venus as observed for Fyn(10)/Gag-Venus. **B**) The three-dimensional reconstruction of distributions of Gag and PIP2 in cells shown in Figure 6A and B. The entire Gag population (left) and the subset of Gag associated with the plasma membrane (defined as Gag located within 1 µm of PIP2) (right) are shown). **C**) Histograms of the distances from PIP2 to Fyn(10)/Gag-Venus in the cells shown in Figure 6A and B. Mean shortest distance values for these two cells are shown with arrowheads. **D**) The mean shortest distances from PIP2 to Fyn(10)/Gag-Venus and Fyn(10)/6A2T/Gag-Venus shown in figure 6C were compared in cells after excluding cells showing very high numbers (>15000) of Fyn(10)/Gag-Venus spots associated with the PM. **E**) the range of Gag-Venus spots compared in panel D. **F**) The cell populations shown in panel 6C were analyzed for PIP2 enrichment as in Figure 5C and D. The experiments were repeated three times, and 9 to thirteen cells for each experiment were analyzed. The *P* values were determined non-paired Student's *t* test. The *P* value was determined using non-paired (Panels D, E and F) or paired (Panel A) Student's *t* test analysis. *, $P< 0.05$ **, $P< 0.01$; ****, $P< 0.0001$; ns, non-significant. Image acquisition, processing, and quantification were performed as in Figures 2-6 . Scale bars, 10 µm.

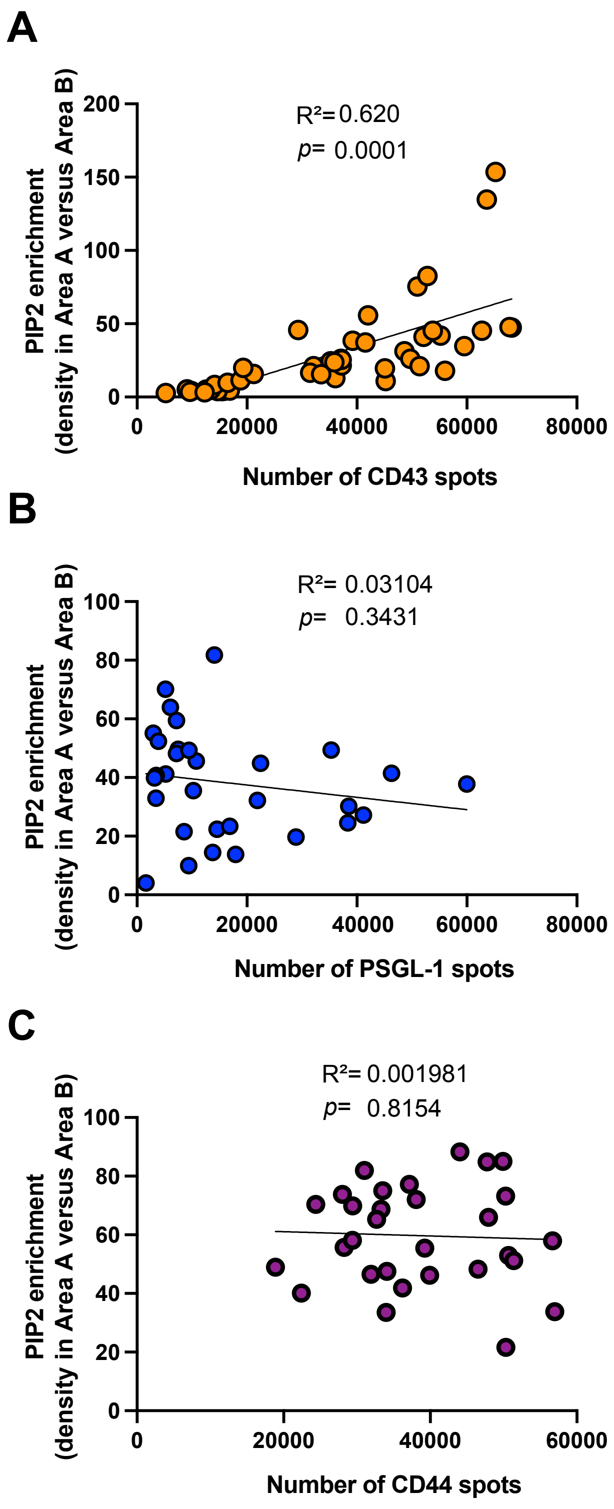
A
R²=0.620
p= 0.0001
PIP2 enrichment
(density in Area A versus Area B)
200
150
100
50
0
0
20000
40000
60000
80000
Number of CD43 spots
B
R²= 0.03104
p= 0.3431
PIP2 enrichment
(density in Area A versus Area B)
100
80
60
40
20
0
0
20000
40000
60000
80000
Number of PSGL-1 spots
C
R²= 0.001981
p= 0.8154
PIP2 enrichment
(density in Area A versus Area B)
100
80
60
40
20
0
0
20000
40000
60000
Number of CD44 spots

Fig. S8.

Analysis of correlation between PIP2 enrichment and plasma membrane signal abundance.
A, **B**, and **C)** Correlations between the number of CD43 WT, PSGL-1, and CD44 spots and PIP2 enrichment in their vicinity are examined. Each dot represents a cell examined in Figure 6 for the PIP2 enrichment. The *P* values and the R^2 are annotated in each corresponding graph.

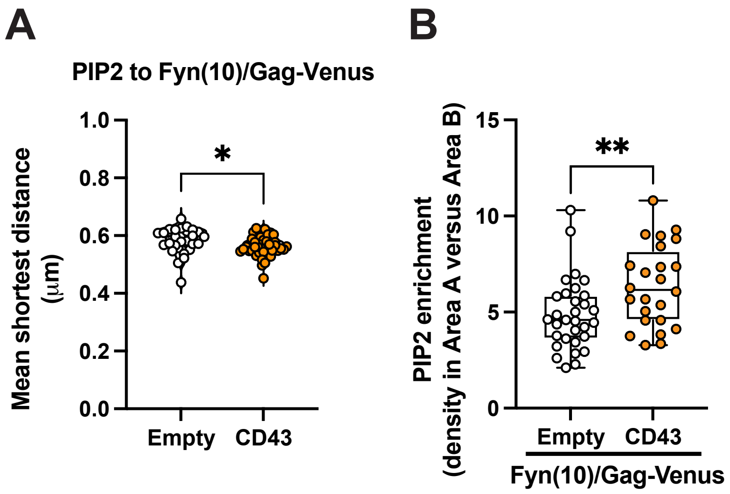
A
PIP2 to Fyn(10)/Gag-Venus
Mean shortest distance
(μm)
1.0
0.8
0.6
0.4
0.2
0.0
*
Empty
CD43
B
PIP2 enrichment
(density in Area A versus Area B)
15
10
5
0
**
Empty
CD43
Fyn(10)/Gag-Venus

Fig. S9.

The effect of the co-expression of CD43 on Gag-PIP2 co-clustering. A and **B**) HeLa cells were transfected with a molecular clone encoding Fyn(10)/Gag-Venus alone or along with the plasmid encoding CD43 WT. The cells were fixed, probed, and expanded as in Figure 5. The means of the shortest distances from PIP2 to Fyn(10)/Gag-Venus (Panel A) and the degree of PIP2 enrichment (Panel B) were determined as in Figure 5. The experiments were repeated three times, and nine to eleven cells from each biological replicate were analyzed. The *P* value was determined using analysis of Student's *t* test. **, $P < 0.01$; *, $P < 0.05$.
